# *Yarrowia lipolytica* Strains Engineered for the Production of Terpenoids

**DOI:** 10.3389/fbioe.2020.00945

**Published:** 2020-08-14

**Authors:** Jonathan Asmund Arnesen, Kanchana Rueksomtawin Kildegaard, Marc Cernuda Pastor, Sidharth Jayachandran, Mette Kristensen, Irina Borodina

**Affiliations:** The Novo Nordisk Foundation Center for Biosustainability, Technical University of Denmark, Kongens Lyngby, Denmark

**Keywords:** terpenes, yeast, metabolic engineering, mevalonate pathway, isoprenoids

## Abstract

Terpenoids are a diverse group of over 55,000 compounds with potential applications as advanced fuels, bulk and fine chemicals, pharmaceutical ingredients, agricultural chemicals, etc. To facilitate their bio-based production, there is a need for plug-and-play hosts, capable of high-level production of different terpenoids. Here we engineer *Yarrowia lipolytica* platform strains for the overproduction of mono-, sesqui-, di-, tri-, and tetraterpenoids. The monoterpene platform strain was evaluated by expressing *Perilla frutescens* limonene synthase, which resulted in limonene titer of 35.9 mg/L and was 100-fold higher than when the same enzyme was expressed in the strain without mevalonate pathway improvement. Expression of *Callitropsis nootkatensis* valencene synthase in the sesquiterpene platform strain resulted in 113.9 mg/L valencene, an 8.4-fold increase over the control strain. Platform strains for production of squalene, complex triterpenes, or diterpenes and carotenoids were also constructed and resulted in the production of 402.4 mg/L squalene, 22 mg/L 2,3-oxidosqualene, or 164 mg/L β-carotene, respectively. The presented terpenoid platform strains can facilitate the evaluation of terpenoid biosynthetic pathways and are a convenient starting point for constructing efficient cell factories for the production of various terpenoids. The platform strains and exemplary terpenoid strains can be obtained from Euroscarf.

## Introduction

**FIGURE 1:**
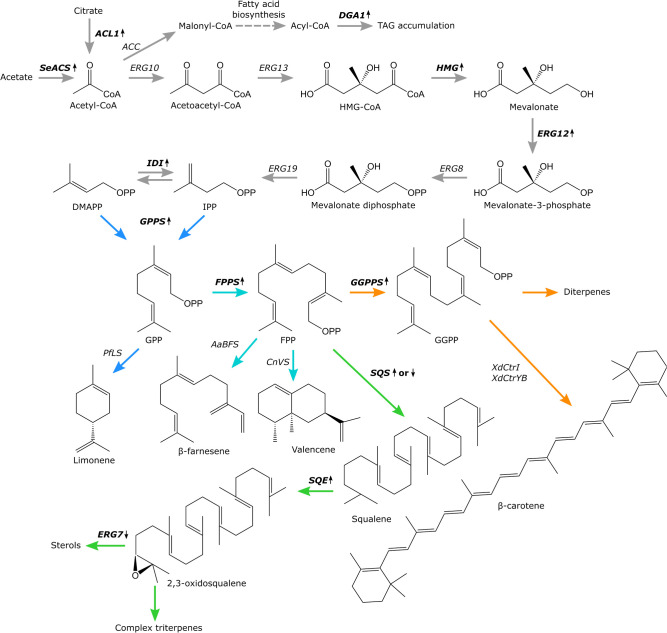
Overview of the MVA-pathway and terpenes produced by engineered *Yarrowia lipolytica* platform strains. Biosynthetic enzymes targeted for upregulation or downregulation are bolded and marked with upward or downward pointing arrows, respectively. *ACL*, ATP citrate lyase 1; *SeACS*, *S. enterica* acetyl-CoA synthetase; *ACC*, acetyl-CoA carboxylase; *ERG10*, acetyl-CoA acetyltransferase; *ERG13*, 3-hydroxy-3-methylglutaryl-CoA synthase; *HMG*, 3-hydroxy-3-methylglutaryl-CoA reductase; *ERG12*, mevalonate kinase; *ERG8*, phosphomevalonate kinase; *ERG19*, mevalonate diphosphate decarboxylase; *IDI*, isopentyl diphosphate isomerase; *GPPS*, geranyl diphosphate synthase; *FPPS* (*ERG20*), farnesyl diphosphate synthase; *GGPPS*, geranylgeranyl diphosphate synthase; *SQS*, squalene synthase; *SQE*, squalene epoxidase; *ERG7*, lanosterol synthase; *PfLS*, *P. frutescens* limonene synthase; *AaBFS*, *A. annua* β-farnesene synthase; *CnVS*, *C. nootkatensis* valencene synthase; *XdCrtI*, *X. dendrorhous phytoene desaturase*; *XdCrtYB*, *X. dendrorhous* bi-functional phytoene synthase/lycopene cyclase. Metabolic branchways are marked with colored arrows, monoterpene biosynthesis, blue. Sesquiterpene biosynthesis, turquoise. Triterpene biosynthesis, green. Diterpene and carotenoid biosynthesis, orange.

Terpenoids comprise the largest class of secondary metabolites; many have biological activity and are used as nutra- and pharmaceutical agents and ingredients for cosmetics or food (Tetali, 2018). Several terpenoids, such as farnesene and bisabolene, have been developed as advanced biofuels. Terpenoids are categorized depending on the number of carbon atoms forming the core skeleton. The commonly studied classes are monoterpenoids (C_10_), sesquiterpenoids (C_15_), diterpenoids (C_20_), triterpenoids (C_30_), and tetraterpenoids (C_40_) also known as carotenoids (Ashour et al., 2010). Other terpenoid classes include hemiterpenoids (C_5_) and sesterterpenoids (C_25_) (Janocha et al., 2015). The core hydrocarbon skeletons assembled from isoprene units are commonly modified by enzymes like cytochromes P450, hydrogenases, methyltransferases, and glycosyltransferases. Terpenoids content in natural sources is typically low and extraction may result in by-products. As an example, the sweet wormwood *Artemisia annua* contains artemisinin, a terpene with anti-malarial properties, but the content is only 0.8% of the plant dry weight (Zyad et al., 2018). Similarly, to extract 1 kg of the flavoring and fragrance ingredient valencene, some sources estimate that 2.5 million kg of oranges is required (Evolva, 2020 Our valencene the natural choice for flavor and fragrance applications). Some terpenoids can be synthesized chemically, but these processes may be extremely complicated. For example, after two decades of research, a full chemical synthesis of azadirachtin, a triterpenoid with potent insect antifeedant properties, was performed with a yield of 0.00015% and no less than 71 steps (Fernandes et al., 2019). Therefore, production of complex terpenoids by chemical synthesis may be too expensive to be economically feasible (Zhang et al., 2017a). Production of terpenoids by fermentation using engineered cell factories can be cheaper and more sustainable than chemical synthesis. Indeed, hosts like the yeast *Saccharomyces cerevisiae* have been engineered to produce a variety of terpenoids and some of these cell factories are currently used industrially (Moser and Pichler, 2019). For example, the sesquiterpene β-farnesene is being produced industrially by highly engineered *S. cerevisiae* with titers of 130 g/L reported in the literature (Meadows et al., 2016). However, since these highly productive, industrial yeast chassis are proprietary, there is a need for accessible microbial terpenoid platform strains. Recently the oleaginous yeast *Yarrowia lipolytica* has attracted attention as a promising host for the production of hydrophobic compounds. The genome of *Y. lipolytica* has been sequenced and convenient toolkits for genetic engineering exists (Dujon et al., 2004; Christen and Sauer, 2011; Holkenbrink et al., 2018; Turck et al., 2019). Furthermore, several *Y. lipolytica* strains have been granted GRAS-status (Groenewald et al., 2014). The species naturally overproduces lipids and hence has a high acetyl-coenzyme A (CoA) flux, which makes it useful for terpenoid production. Terpenoids like limonene, linalool, α-farnesene, betulinic acid, and β-carotene have previously been produced in recombinant *Y. lipolytica* strains (Yang et al., 2016; Cao et al., 2017; Gao et al., 2017; Cheng et al., 2019; Jin et al., 2019). In yeasts, the C_5_-precursor of terpenoids isopentyl diphosphate (IPP), is produced via the cytosolic mevalonate (MVA) pathway (Figure 1; Ashour et al., 2010; Cao et al., 2016). The initial steps of the MVA-pathway start with the condensation of three acetyl-CoA molecules forming 3-hydroxy-3-methylglutaryl-CoA (HMG-CoA). Subsequently, HMG-CoA is reduced to mevalonic acid, which is then phosphorylated twice and lastly decarboxylated, forming IPP which can isomerize to form dimethylallyl diphosphate (DMAPP). These phosphorylated C_5_-precursors can condensate to form geranyl diphosphate (GPP), farnesyl diphosphate (FPP), geranylgeranyl diphosphate (GGPP) that then generate the backbones of monoterpenoids, sesqui- and triterpenoids, or diterpenoids and carotenoids, respectively. Although *Y. lipolytica* may be highly suited for the production of terpenoids, the yeast strains often require several cycles of engineering and optimization before high terpenoid titers can be reached. Since terpenoids are derived from the same basic IPP/DMAPP-building blocks, pre-engineered “platform” strains with high MVA-pathway flux could be engineered (Cravens et al., 2019). Upon integration of terpenoid biosynthetic genes, such platform strain could provide immediately improved titers, which would shorten the downstream engineering process considerably. Furthermore, platform strains tailored toward the production of specific terpenoid classes could be constructed, since the subsequent pathways from IPP/DMAPP diverge toward mono-, sesqui-, tri-, or diterpenoids and carotenoids. Therefore, this study aimed to develop pre-engineered platform strains of *Y. lipolytica* with improved production of GPP for monoterpene production, FPP for sesquiterpene production, squalene/2,3-oxidosqualene for triterpene production, or GGPP for diterpenoid and carotenoid production. While these platform strains require additional engineering before industrially relevant titers can be reached, they can serve as convenient plug-and-play hosts for testing new or improved enzymes toward various terpenoids and for testing new metabolic engineering strategies for improving terpenoid production in *Y. lipolytica*. By making the strains available via Euroscarf, we hope to initiate a scientific community effort of building superior platform strains together.

## Materials and Methods

### Yeast Strains and Media

The W29-derived ST6512 (MatA ku70Δ:PrTEF1->Cas9-TTef12:PrGPD->DsdA-TLip2) expressing Cas9 for CRISPR/based DNA integration was used to construct the platform strains (Marella et al., 2019). This strain was based on the W29 *Y. lipolytica* (MatA) strain Y-63746, which was a kind gift from the ARS Culture Collection, NCAUR, United States. YPD-media containing 10 g/L yeast extract, 20 g/L peptone, and 20 g/L glucose were used to grow the *Y. lipolytica* strains at 30°C. 20 g/L agar was added for solid media. For selection, either nourseothricin (250 mg/L) or hygromycin (400 mg/L) was added to the media. Cultivation of strains for terpenoid production was done in YP-media with 80 g/L glucose. *Escherichia coli* strain DH5α was used for plasmid construction. The *E. coli* cells were cultivated at 37°C on Lysogeny Broth (LB) medium supplemented with 100 mg/L ampicillin for plasmid selection. The chemicals were obtained, if not indicated otherwise, from Sigma-Aldrich. Nourseothricin was purchased from Jena BioScience GmbH (Germany).

### DNA Constructs

The primers, biobricks, plasmids, and primers used in this study are listed in Supplementary Tables 1–4, respectively. The biobricks were amplified with PCR using Phusion U polymerase (Thermo Scientific) and assembled into the EasyCloneYALI vectors with USER cloning (Holkenbrink et al., 2018). The USER reactions were transformed into *E. coli* and correct assemblies were verified by sequencing. Codon-optimized genes encoding *Citrus limon* limonene synthase (*ClLS*) (Accession: Q8L5K3.1), *Perilla frutescens* limonene synthase (*PfLS*) (Accession: AJW68081.1), *Artemisia annua* β-farnesene synthase (*AaBFS*) (MT276895.1), *Callitropsis nootkatensis* valencene synthase (*CnVS*) (Accession: AFN21429.1), *Xanthophyllomyces dendrorhous* phytoene desaturase (*XdCrtI*) (Accession: ATB19150.1), *X. dendrorhous* bi-functional phytoene synthase/lycopene cyclase (*XdCrtYB*) (Accession: Q7Z859.1) with an A190T substitution, and *Salmonella enterica* acetyl-CoA synthetase (*SeACS*) (Accession: WP_000083882.1) with a L641P substitution as described in Huang et al. (2018) were ordered as GeneArt String DNA fragments from Thermo Fischer Scientific. The codon-optimized nucleotide sequences can be found the Supplementary Material.

*ERG20* was mutated into *ERG20^*F*88*C*^* based on an alignment of *Y. lipolytica* Erg20p (Accession number: XP_503599.1) and *S. cerevisiae* Erg20p (Accession number: CAA89462.1) amino sequences (Supplementary Figure 1). The mutated residue was selected based on a previous report (Ignea et al., 2015). The alignment was made using Benchling software and was visualized with MView 1.63 (Benchling, 2020 Cloud-Based Informatics Platform for Life Sciences R&D | Benchling; Brown et al., 1998). In addition, *ERG20* was mutated into *ERG20^*F*88*W–N*119*W*^* based on a previous report (Cao et al., 2017). The amino acid substitutions were constructed with PCR mutagenesis and the primers are listed in Supplementary Table 4.

### Yeast Transformation

The yeast strains used in this study are listed in Supplementary Table 1. A lithium-acetate based transformation protocol as described previously was used for yeast strain engineering and integration vectors were linearized with *Not*I-enzyme before transformation (Holkenbrink et al., 2018). Correct integration was verified with colony PCR using vector-specific primers and primers complementary to the genomic region adjacent to the integration site.

### Yeast Cultivation

Yeast strains were inoculated into 2.5 mL YPD in 24-well plates with air-penetrable lid (EnzyScreen, Netherlands) and grown for 16–24 h at 30°C and 300 rpm agitation at 5 cm orbit cast. Then for limonene, β-farnesene, and valencene, the required volume for a starting 600 nm optical density (OD) of 0.1 was transferred to 2 mL of YP with 80 g/L glucose in glass tubes and a dodecane overlay of 200 μL was added. For squalene, 2,3-oxidosqualene, and β-carotene, the required volume for a starting 600 nm optical density (OD) of 0.1 was transferred to 2.5 mL of YP with 80 g/L glucose in a 24-deepwell plate. The inoculated media was incubated for 72 h at 30°C with 300 rpm agitation. All cultivations were performed in biological triplicates. OD and cell dry weight was measured by the end of cultivation.

### Metabolite Analysis

For limonene, β-farnesene, and valencene, the cell culture broth was centrifuged and the dodecane phase was harvested for analysis. The dodecane samples were diluted in cyclohexane with either patchoulol or myrcene as an internal standard for valencene and β-farnesene, or limonene, respectively. β-carotene, was extracted as previously described (Kildegaard et al., 2017). For squalene and 2,3-oxidosqualene, 1 mL of culture broth was transferred into a 2 mL microtube, centrifuged and the supernatant was discarded. 500 μL of 0.5–0.75 mm acid-washed glass beads and 1 mL of acetonitrile were added to each sample. The samples were then incubated at 95°C at 650 rpm shaking for 1 h in a Thermo-Shaker TS-100 (biosan). Subsequently, the cells were broken with a Precellys^®^24 homogenizer (Bertin Corp.) four times at 5500 rpm for 20 s with samples being kept on ice in between rounds of breaking. Lastly, the samples were centrifuged, and the supernatant was collected for analysis.

Gas chromatographic (GC) analysis for valencene, limonene, and β-farnesene was conducted using Thermo Scientific GC Trace 1300 equipment with a flame ionization detector (FID) and equipped with a fused-silica capillary column (BPX5, 30 m × 0.25 mm ID, 0.25 μm, SGE Analytical Science). Helium at a constant flow rate of 1.0 mL/min was used as the carrier gas. The GC oven temperature started at 50°C for 1.5 min and then increased to 170°C at 30°C/min and hold for 1.5 min. Then from 170 to 300°C at 15°C/min and hold for 4.5 min, finalizing the chromatographic run at 20.2 min. The injector and detector ports were both kept at 300°C and the injector operated in a split mode of 20:1. Concentrations of beta-carotene, squalene, and 2,3-oxidosqualene were measured using a Dionex Ultimate 3000 HPLC with a Supelco Discovery HS F5-3 HPLC column (150 × 2.1 mm, 3 μm particle size) and a DAD-3000 Diode Array Detector at 450 and 210 nm, respectively. The mobile phase consisted of A: 10 mM ammonium formate and B: acetonitrile. The flow rate was 0.7 mL/min and the column was kept at 30°C. The mobile phase was introduced as 25% B and held at this composition for 2 min. The gradient was then ramped to 90% B to 4 min and this gradient held to 10.5 min and followed by a linear gradient to 25% B at 11.25 min. The column was equilibrated with 25% B until 13.5 min. Samples were held at 5°C during the analysis and 1 μL sample volume injected. Data analysis was performed using Chromeleon 7.2.9 and the analyte quantification was performed using peak area ratios of authentic standards. Quantities of limonene for ST9249, ST9395, and squalene for ST6512 were extrapolated from the standard curve regression.

## Results

### Design of *Y. lipolytica* Platform Strains for Terpenoid Production

To design the platform strains, we compiled a list of studies for terpenoid production in *Y. lipolytica* (Table 1). While some studies generated highly modified strains by, for example, overexpressing the entire MVA-pathway, it may not be clear from the experimental design, which exact modifications had resulted in improvement of terpenoid titers. Therefore, we selected metabolic engineering strategies that had been clearly demonstrated to result in improved terpenoid production. These strategies formed the basis of the platform strains. Two key strategies were implemented for all platform strains: improvement of the precursor acetyl-CoA pool and up-regulation of the MVA-pathway to improve the accumulation of IPP/DMAPP (Figure 1). Increasing the acetyl-CoA pool was done by overexpression of the native ATP citrate lyase 1 (*ACL*) and the *Salmonella enterica* acetyl-CoA synthetase (*SeACS*), as this was shown to increase squalene titers in *Y. lipolytica* in combination with overexpression of the native 3-hydroxy-3-methylglutaryl-CoA reductase (*HMG*) (Huang et al., 2018). In *Y. lipolytica*, Aclp generates acetyl-CoA and oxaloacetate from citrate, whereas SeAcsp produces acetyl-CoA from acetate and CoA. To increase MVA-pathway flux, several genes involved in this pathway were overexpressed. The formation of mevalonic acid from 3-hydroxy-3-methylglutaryl-CoA is catalyzed by Hmgp and this is considered a key limiting step in the MVA-pathway (Ashour et al., 2010). Overexpression of *HMG* has been shown to boost the production of α-farnesene, linalool and limonene in *Y. lipolytica* (Cao et al., 2016, 2017; Yang et al., 2016). Although truncated versions of Hmgp have been used in *S. cerevisiae*, studies indicate that the non-truncated version is superior for terpenoid production in *Y. lipolytica* (Cao et al., 2016; Kildegaard et al., 2017; Bröker et al., 2018; Han et al., 2018). For example, the production of β-carotene was enhanced to a greater degree by *HMG* compared to *tHMG* both when solely expressed or in combination with the geranylgeranyl diphosphate synthase (*GGPPS*) (Kildegaard et al., 2017). Indeed, non-truncated *HMG* has also been used for production of several other terpenoids in *Y. lipolytica* such as betulinic acid (Table 1; Jin et al., 2019). Furthermore, overexpression of the mevalonate kinase (*ERG12*) in combination with *HMG* was shown to increase limonene titers in *Y. lipolytica* (Cao et al., 2016). Lastly, overexpression of the isopentyl diphosphate isomerase (*IDI*) that catalyzes the isomerization of IPP and DMAPP together with *HMG* has been shown to increase the production of linalool and α-farnesene in *Y. lipolytica* (Yang et al., 2016; Cao et al., 2017). Therefore, overexpression of *HMG*, *ERG12*, and *IDI* was selected to form the basis of the terpenoid platform strains. To further tailor the platform strains toward the production of either mono-, sesqui-, tri- or diterpenoids and carotenoids, strategies were implemented to direct IPP/DMAPP toward the appropriate phosphorylated isoprene unit and prevent the flux of this substrate toward undesired products. Monoterpenoids are derived from the GPP which is formed by the condensation of DMAPP and IPP (Ignea et al., 2014). Mutating the phenylalanine residue in position 96 and the asparagine residue in position 127 to tryptophan of *S. cerevisiae* Erg20p changed its function to a geranyl diphosphate synthase (Ignea et al., 2014). A *Y. lipolytica* Erg20p version with identical mutations in the positionally similar residues (*ERG20^*F*88*W–N*119*W*^*) was used for linalool production in *Y. lipolytica* (Cao et al., 2017). Therefore, the mutated *Y. lipolytica* variant was selected to direct the flux of IPP/DMAPP toward monoterpenoid production. Both sesquiterpenoids and triterpenoids use FPP as the starting substrate, with FPP being dephosphorylated and typically re-arranged to form sesquiterpene backbones, while two units of FPP dimerize to form squalene, which is a precursor for many triterpenoids and sterols. Therefore, the native farnesyl diphosphate synthase (*ERG20*) was overexpressed in the sesqui- and triterpenoid platform strains, which also had been used to increase nootkatone production in *Y. lipolytica* (Guo et al., 2018). Furthermore, since complex, cyclical triterpenoids often derive from 2,3-oxidosqualene, the squalene synthase (*SQS*) and squalene epoxidase (*SQE*) were overexpressed in the triterpenoid platform strain to increase 2,3-oxidosqualene formation. The substrate for diterpenoids and carotenoids is GGPP. Therefore, either the geranylgeranyl diphosphate synthase (*GGPPS*) or a mutated version of the native Erg20p, where the phenylalanine residue in position 96 was changed to tryptophan (*ERG20^*F*88*C*^*), were overexpressed to generate two different diterpene/carotenoid platform strains (Supplementary Figure 1). A similar mutation in the *S. cerevisiae* Erg20p was demonstrated to change its function to a geranylgeranyl diphosphate synthase and improve diterpene production in *S. cerevisiae* (Ignea et al., 2015). To prevent carbon loss to undesired products, we sought to downregulate squalene formation in the mono-, sesqui-, diterpenoid and carotenoid platform strains by exchanging the native squalene synthase promoter (*pERG9*) with the relatively weak lanosterol 14-α-demethylase promoter (*pERG11*). Squalene is essential for sterol production which necessitates downregulation rather than deletion of *SQS* and the former strategy had previously been shown to increase β-carotene production in *Y. lipolytica* (Kildegaard et al., 2017). For the triterpene platform strain, the native lanosterol synthase promoter (*pERG7*) was truncated to decrease the flux of 2,3-oxidosqualene toward sterol synthesis. Both truncations leaving either a 100 or 50 remaining base pairs (bp) of the 3′ end of *pERG7* were constructed. By implementing these four strategies, platform strains for either mono-, sesqui-, tri-, or diterpene/carotenoids were constructed.

**TABLE 1:** Metabolic engineering of *Yarrowia lipolytica* for terpenoid biosynthesis.

Compound	Carbon Source	Parental Strain	Relevant modifications related to terpenoid biosynthesis	Titer	References
**Monoterpenoids**					
Limonene	Glucose	Po1g	↑***HMG*** (↑***ClLS*** or ↑*MsLS*)	D-limonene: 11.7 mg/L L-limonene: 11.1 mg/L (bioreactor)	Pang et al., 2019
	Glycerol Citrate	Po1f	↑*ArtLS* ↑*SltNDPS1* ↑***HMG1*** ↑***ERG12***	165.3 mg/L (bioreactor)	Cheng et al., 2019
	Glucose Pyruvic acid	Po1f	↑*ArtLS* ↑*SltNDPS1* ↑***HMG1*** ↑***ERG12***	23.6 mg/L (shake flask)	Cao et al., 2016
	Glucose	ATCC 20460	↑***HMG1*** ↑***ERG12*** ↑***ACL1*** ↑***SeACS*** ↑***IDI*** ↑***ERG20^*F*88*W–N*119*W*^*** ↓***SQS*** ↑***PfLS***	35.9 mg/L (glass tube)	This study
Linalool	Citrate Pyruvate	Po1f	↑*AaLIS* ↑***ERG20^*F*88*W–N*119*W*^*** ↑***HMG*** ↑***IDI***	6.96 mg/L (shake flask)	Cao et al., 2017
**Sesquiterpenoids**					
α-farnesene	Glucose Fructose	Po1h	↑*SctHMG* ↑***IDI*** ↑*MdFS*-*L*-*ERG20*	259.98 mg/L (bioreactor)	Yang et al., 2016
	Glucose	Po1f	↑*BdHMG* ↑*ERG13* ↑*MdFS-L-ERG20* ↑***ERG12*** ↑***IDI*** ↑*ERG8*,19 ↑***GPPS***	25.55 g/L (bioreactor)	Liu et al., 2019
β-farnesene	Glucose	ATCC 20460	↑***HMG1*** ↑***ERG12*** ↑***ACL1*** ↑***SeACS*** ↑***IDI*** ↑***ERG20*** ↑***AaBFS***	955 mg/L (glass tube)	This study
β-ionone	Glucose	Po1f	↑*carB* ↑*carRP* ↑*PhCCD1* ↑***GGPPS*** ↑*tHMG* ↑***ERG****8,10*,***12****,13,19,20*Δ*pox3,5* ↑***IDI*** ↑*bbPK* ↑*bsPTA*	0.98 g/L (bioreactor)	Lu et al., 2020
	Glucose	Po1f	↑*carB* ↑*carRP* ↑*OfCCD1* ↑*SsNphT7* ↑*HpIDI* ↑***ERG****8,10*,***12****,13,19* ↑*tHMG1* ↑***GPPS*** ↑*ERG20*-*GGPPS*	380 mg/L (bioreactor)	Czajka et al., 2018
α-santalene	Glucose	ATCC 201249	↑*ClSTS* ↑*ERG8* ↑***HMG***	27.92 mg/L (bioreactor)	Jia et al., 2019
Nootkatone valencene	Glucose	ATCC 201249	↑***CnVS*** ↑*CnCYP706M1-AtATR1* ↑*tHMG* ↑***ERG20***	Nootkatone: 978.2 μg/L Valencene: 22.8 mg/L (shake flask)	Guo et al., 2018
Valencene	Glucose	ATCC 20460	↑***HMG1*** ↑***ERG12*** ↑***ACL1*** ↑***SeACS*** ↑***IDI*** ↑***ERG20*** ↓***SQS*** ↑***CnVS***	113.9 mg/L (glass tube)	This study
Amorphadiene	Glucose	Po1g	↑*AaADS* ↑***HMG1*** ↑***ERG12***	171.5 mg/L (shake flask)	Marsafari and Xu, 2020
**Triterpenoids**					
Campesterol	Sunflower seed oil	ATCC 201249	Δ*ERG5* ↑*XlDHCR7*	453 mg/L (bioreactor)	Du et al., 2016
	Sunflower seed oil	ATCC 201249	Δ*ERG5* ↑*DrDHCR7* ↑*POX2*	942 mg/L (bioreactor)	Zhang et al., 2017b
Ginsenoside K	Glucose	ATCC 201249	↑*tHMG* ↑***ERG20*** ↑***SQS*** ↑*PgDS* ↑*PgPPDS* ↑*AtATR1* ↑*PgUGT1*	161.8 mg/L (bioreactor)	Li et al., 2019
Oleanolic Acid	Glucose	ATCC 201249	↑*tHMG* ↑***ERG20*** ↑***SQS*** ↑*GgBAS* ↑*MtCYP716A12-L-AtATR1*	540.7 mg/L (bioreactor)	Li et al., 2020
Betulinic acid	Glycerol	ATCC 201249	↑*tHMG1* ↑***SQS*** ↑*AtLUP1* ↑*MtCYP716A12* ↑*AtATR1*	26.53 mg/L (shake flask)	Sun et al., 2019
	Glucose	ATCC 201249	↑*RcLUS* ↑*BPLO* ↑*LjCPR* ↑***SQS*** ↑***SQE*** ↑***HMG1*** ↑*MFE1*	204.89 mg/L (shake flask)	Jin et al., 2019
Squalene	Glucose Citrate	Po1f	↑***HMG1*** ↑***ACL1*** ↑***SeACS***	10 mg/gDCW (shake flask)	Huang et al., 2018
	Glucose	ATCC MYA2613	↑*carB* ↑*carRP* ↑***ERG****8,10*,***12****,13,19*,***20*** ↑*tHMG* ↑***IDI*** Δ*gut2* Δ*pox3,4,5,6*	531.6 mg/L	Gao et al., 2017
	Glucose	ATCC 20460	↑***HMG1*** ↑***ERG12*** ↓***ERG7*** ↑***ACL1*** ↑***SeACS*** ↑***IDI*** ↑***ERG20*** ↑***SQS***	402.4 mg/L (deepwell plate)	This study
2,3-oxidosqualene	Glucose	ATCC 20460	↑***HMG1*** ↑***ERG12*** ↓***ERG7*** ↑***ACL1*** ↑***SeACS*** ↑***IDI*** ↑***ERG20*** ↑***SQS*** ↑***SQE***	22 mg/L (deepwell plate)	This study
Protopanaxadiol	Xylose	ATCC 201249	↑*SsXYL1* ↑*SsXYL2* ↑*XKS* ↑*PgDS* ↑*PgPPDS-L-AtATR1* ↑*tHMG* ↑***ERG20*** ↑***SQS*** ↑*TKL* ↑*TAL* ↑*TX*Δ*pox1,2,3*	300.63 mg/L (bioreactor)	Wu et al., 2019
**Carotenoids**					
Lycopene	Glucose	H222	↑*PaCrtB* ↑*PaCrtI* ↑***GGPPS*** ↑***HMG1*** Δ*pox1–6* Δ*gut2*	16 mg/gDCW (bioreactor)	Matthäus et al., 2014
	Glucose	Po1f	↑***HMG*** ↑*PaCrtE* ↑*PaCrtB* ↑*PaCrtI* ↑*ERG19*	21.1 mg/gDCW (bioreactor)	Schwartz et al., 2017
β-Carotene	Glucose	ATCC MYA2613	↑*carB* ↑*carRP* ↑***ERG****8,10*,***12***, *13,19*,***20*** ↑***GGPPS*** ↑*tHMG* ↑***IDI*** Δ*pox3,4,5,6*	4 g/L (bioreactor)	Gao et al., 2017
	Glucose	Po1f	↑*carB* ↑*carRP* ↑***GGPPS*** ↑***HMG*** ↑*ERG13* Δ*pox2,3*	4.5 g/L (bioreactor)	Zhang et al., 2020
	Glucose	ATCC 20460	↑*carB* ↑*carRP* ↑***HMG*** ↑***GGPPS*** ↑*DGA2* ↑*GPD1*Δ*pox1–6* Δ*tgl4*	6.5 g/L (bioreactor)	Larroude et al., 2018
	Glucose	ATCC 20460	↑***HMG1*** ↑***ERG12*** ↑***ACL1*** ↑***SeACS*** ↑***IDI*** ↑**GGPPS**↓***SQS*** ↑***XdcrtYB*** ↑***XdcrtI***	164 mg/L (deepwell plate)	This study
Astaxanthin	Glucose	GB20	↑***XdcrtYB*** ↑***XdcrtI*** ↑***HMG*** ↓***SQS*** ↑*XdcrtE* ↑*PscrtW* ↑*PacrtZ*	54.6 mg/L (microtiter plate)	Kildegaard et al., 2017
	Glucose	GB20	↑***XdcrtYB*** ↑***XdcrtI*** ↑***HMG*** (↑SQS ↑XdcrtE ↑SsGGP*PS* ↑*HpBKT* ↑*HpCrtZ*	285 mg/L (bioreactor)	Tramontin et al., 2019

*Genes modified in this study are bolded in the table.** AaADS**, Artemisia annua amorphadiene synthase;** AaBFS**, A. annua β-farnesene synthase;** AaLIS**, Actinidia arguta linalool synthase; **ACL1**, ATP citrate lyase 1;** ArtLS**, Agastache rugosa limonene synthase; **AtATR1**, Arabidopsis thaliana NADPH-cytochrome P450 reductase 1; **AtLUP1**, A. thaliana lupeol synthase; **BbPK**, Bifidobacterium bifidum phosphoketolase; **BdHMG**, Bordetella petrii HMG; **BpLO**, Betula platyphylla lupeol C-28 oxidase; **BsPTA**, Bacillus subtilis phosphotransacetylase; **carB**, Mucor circinelloides phytoene dehydrogenase; **carRP**, M. circinelloides phytoene synthase/lycopene cyclase;** CnCYP706M1-AtATR1**, fusion of Callitropsis nootkatensis cytochrome P450 and AtATR1; **CnVS**, C. nootkatensis valencene synthase; **ClLS**, Citrus limon d-limonene synthase; **ClSTS**, Clausena lansium α-santalene synthase; **DGA2**, diacylglycerol acyltransferase 2; **DrDHCR1**, Danio rerio 7-dehydrocholesterol reductase; **ERG5**, C22-sterol desaturase; **ERG7**, lanosterol synthase; **ERG8**, phosphomevalonate kinase gene;** ERG10**, acetyl-CoA C-acetyltransferase; **ERG12**, mevalonate kinase; **ERG13**, Hydroxymethylglutaryl-CoA synthase;** ERG19**, mevalonate diphosphate decarboxylase encoding gene;** ERG20**, farnesyl diphosphate synthase; **ERG20^F88W–N119W^**, geranyl diphosphate synthase; **ERG20-GGPPS**, fusion of ERG20 and **GGPPS**, geranylgeranyl diphosphate synthase;** GgBAS**, Glycyrrhiza glabra β-amyrin synthase; **GPD**, glyceraldehyde-3-phosphate dehydrogenase; **GPPS**, geranyl diphosphate synthase; **HMG**, Hydroxymethylglutaryl-CoA reductase; **HpBKT**, Haematococcus pluvialis β-carotene ketolase; **HpIPI**, Haematococcus pluvialis isopentyl diphosphate isomerase; **HpCrtZ**, H. pluvialis β-carotene hydroxylase; **IDI**, isopentyl diphosphate isomerase; **LjCPR**, Lotus japonicus cytochrome P450 reductase; **MdFS-L-ERG20**, Malus domestica α-farnesene synthase linked to ERG20;** MFE1**, multifunctional β-oxidation enzyme 1; **MsLS**, Mentha spicata l-limonene synthase;** MtCYP716A12-L-AtATR1**, Medicago truncatula cytochrome P450 fused to AtATR1; **OfCCD1**, Osmanthus fragrans carotenoid cleavage dioxygenase 1; **PaCrtB**, Pantoea ananatis phytoene synthase;** PaCrtE**, P. ananatis geranylgeranyl diphosphate syntase; **PaCrtI**, P. ananatis phytoene desaturase; **PacrtZ**, P. ananatis β-carotene hydroxylase; **PfLS**, P. frutescens limonene synthase;** PgDS**, Panax ginseng dammarenediol II synthase; **PgPPDS**, P. ginseng cytochrome P450 enzyme;** PgPPDS-L-AtATR1**, PgPPDS linked to AtATR1;** PgUGT1**, P. ginseng UDP-glycosyltransferase; **PhCCD1**, Petunia hybrida carotenoid cleavage dioxygenase; **POX**, peroxisome acyl-CoA oxidase; **PscrtW**, Paracoccus sp. N81106 β-carotene ketolase; **RcLUS**, Ricinus communis lupeol synthase; **SctHMG**, Saccharomyces cerevisiae truncated HMG, **SeACS**, S. enterica Acetyl-CoA synthetase; **SQE**, squalene epoxidase; **SQS**, squalene synthase; **SlNDPS1**, Solanum lycopersicum neryl diphosphate synthase 1;** SsGGPPS**, Synechococcus sp. GGPPS; **SsNphT7**, Streptomyces sp. Acetoacetyl CoA synthase; **SsXYL1**, Scheffersomyces stipitis xylose reductase XR; **SsXYL2**, S. stipitis xylose reductase XDH; **TAL**, transaldolase; **TGL**, triacylglycerol lipase;** TKL**, transketolase;** TX**, xylose transporter; **XdcrtE**, X. dendrorhous geranylgeranyl diphosphate synthase;** XdcrtI**, X. dendrorhous phytoene desaturase; **XdcrtYB**, Xanthophyllomyces dendrorhous bi-functional phytoene synthase/lycopene cyclase; **XKS**, xylulose kinase; **XlDHCR7**, Xenopus laevis 7-dehydrocholesterol reductase.*

### Evaluation of the Metabolic Engineering Strategies Using β-Farnesene as the Test Case

**FIGURE 2:**
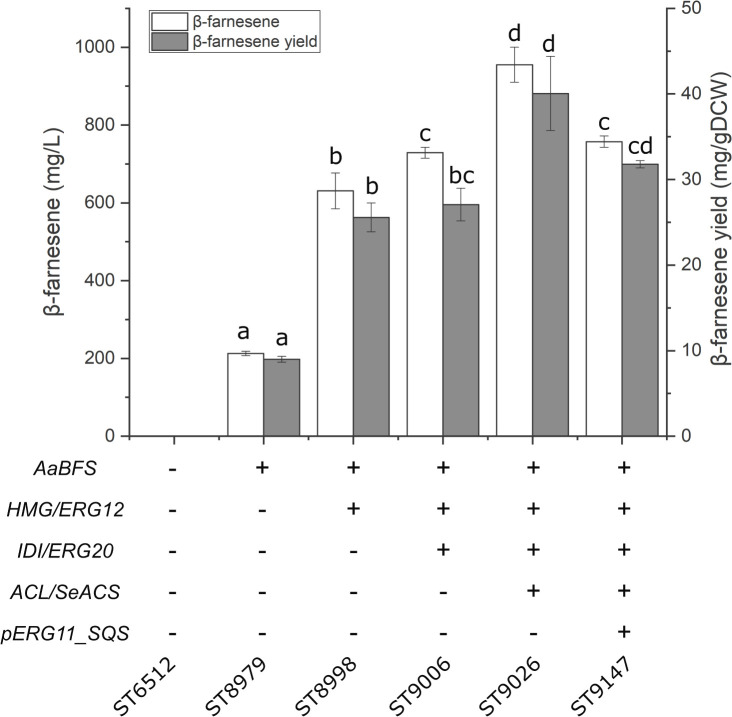
β-farnesene production by the sesquiterpene platform strain. The cumulative effect of engineering strategies for β-farnesene production. *AaBFS*, *A. annua* β-farnesene synthase; *HMG*, HMG-reductase; *ERG12*, mevalonate kinase; *IDI*, isopentyl diphosphate isomerase; *ERG20*, farnesyl diphosphate synthase; *ACL*, ATP citrate lyase 1; *SeACS*, *S. enterica* acetyl-CoA synthetase; *pERG11*_*SQS*, substitution of the native squalene synthase promoter with the *pERG11*-promoter. Standard deviations from the values of three biological replicates are represented by error bars. Statistical significance (students *t*-test, two-tailed, *p* < 0.05) is denoted by the letters a, b, c, and d.

The selected metabolic engineering strategies were evaluated using sesquiterpene β-farnesene as the test compound. The strategies were then combined to generate platform strains without heterologous terpenoid pathways. β-farnesene is an acyclic sesquiterpene with potential applications as a biofuel precursor (George et al., 2015). Furthermore, β-farnesene has been produced at high titers in *S. cerevisiae*, which makes it an excellent compound for testing metabolic engineering strategies (Meadows et al., 2016). Therefore, the β-farnesene synthase from *Artemisia annua* (*AaBFS*) was integrated into the *Y. lipolytica* genome under expression of the *pTEFintron* promoter. This test-strain solely expressing *AaBFS* produced 212.7 ± 5.6 mg/L β-farnesene (Figure 2). Subsequently, the strategies for improvement of sesquiterpene production were implemented to discern their effect on β-farnesene production. The strategies were performed consecutively on each newly generated strain to demonstrate the cumulative effect of the modifications. First, the native *Y. lipolytica* genes *HMG* and *ERG12* were overexpressed in the β-farnesene producing strain, which increased β-farnesene titers to 631 ± 46.1 mg/L, an approximately 3-fold increase compared to the strain solely expressing *AaBFS*. Subsequently, overexpression of *IDI* and *ERG20* resulted in 729 ± 13.8 mg/L β-farnesene. Thirdly, the genes *ACL* and *SeACS* were expressed, which raised the titer to 955 ± 45.1 mg/L, a 4.5-fold increase compared to the sole expression of *AaBFS.* Lastly, the native squalene promoter was replaced with the weak promoter *pERG11* (*pERG11_SQS*). However, promoter replacement reduced the β-farnesene titer to 757 ± 14.5 mg/L.

### Monoterpene Platform Strain

**FIGURE 3:**
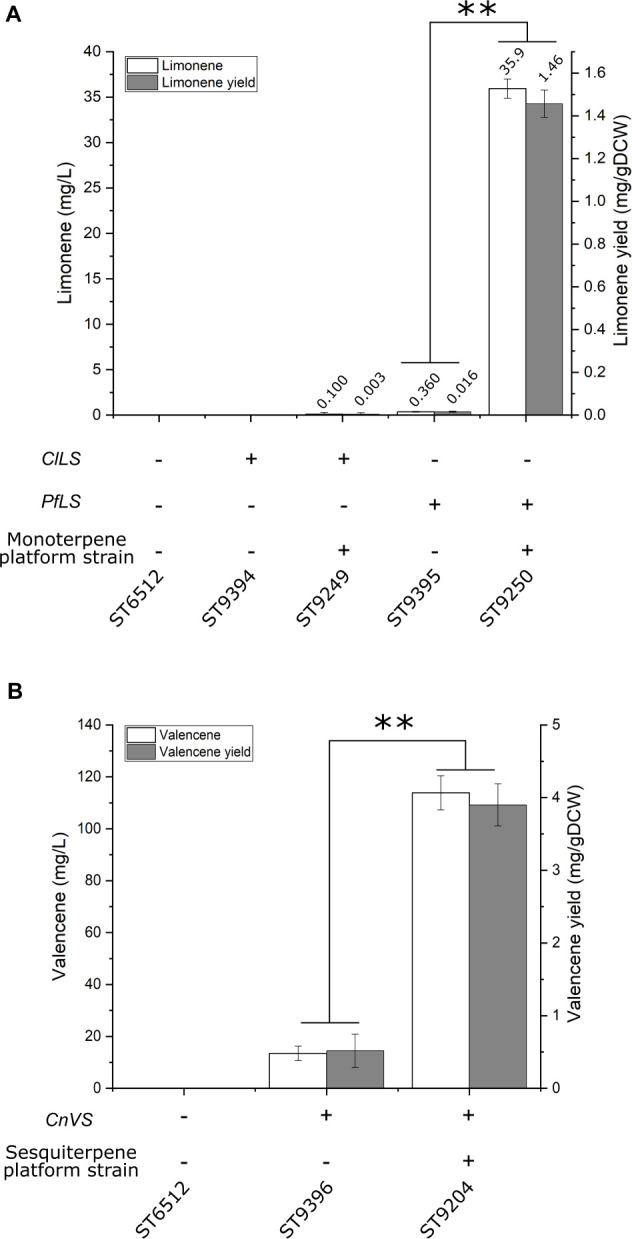
Limonene and valencene production by platform strains. **(A)** Limonene production in base and monoterpene platform strain. *ClLS*, *C. lemon* limonene synthase; *PfLS*, *P. frutescens* limonene synthase. The monoterpene platform strain contains the following gene overexpressions of *HMG*, *ERG12*, *ACL*, *SeACS*, *IDI, ERG20^*F*88*W–N*119*W*^*, and swapping the native squalene promoter (*pERG11_SQS*). **(B)** Valencene production in base and sesquiterpene platform strain. *CnVS*, *C. nootkatensis* valencene synthase. The sesquiterpene platform strain contains the same modifications as the monoterpene platform strain, with the exception of *ERG20* instead of *ERG20^*F*88*W–N*119*W*^*. Standard deviations from the values of three biological replicates are represented by error bars. Double asterisks marks significant change (student’s *t*-test, two-tailed, *p* < 0.001).

A platform strain for monoterpene production was constructed by expression of *HMG*, *ERG12*, *ACL*, *SeACS*, *IDI, ERG20^*F*88*W–N*119*W*^*, and swapping the native squalene promoter (*pERG11_SQS*). To test the potential of the monoterpene platform strain, either a limonene synthase from *Perilla frutescens* (*PfLS*) or *Citrus limon* (*ClLS*) was expressed in the platform strain and the base strain. Limonene is a cyclic monoterpene used extensively as a flavor and fragrance ingredient due to its citrus-like characteristics (Cao et al., 2016). No limonene was detected for the base strain expressing *ClLS* (Figure 3A). The monoterpene platform strain expressing *ClLS* produced 0.1 ± 0.2 mg/L limonene. The base strain expressing *PfLS* produced 0.36 ± 0.04 mg/L limonene, while the monoterpene platform strain expressing *PfLS* produced 35.9 ± 1.1 mg/L limonene. This represents an almost 100-fold increase in limonene titer compared to the base strain.

### Sesquiterpene Platform Strain

In parallel with the β-farnesene producing test-strain, a sesquiterpene platform strain was generated with the same modifications except for the integration and expression of *AaBFS*. This sesquiterpene platform strain was tested by expression of a valencene synthase from *Callitropsis nootkatensis* (*CnVS*) generating strain ST9204. Valencene is a cyclic sesquiterpene with a citrus-like aroma used for flavoring or fragrances (Beekwilder et al., 2014). For comparison, *CnVS* was expressed in a strain without any improvements generating strain ST9396. The sesquiterpene platform strain ST9204 produced 113.9 ± 6.6 mg/L valencene, while ST9396 produced 13.5 ± 2.8 mg/L (Figure 3B). This corresponds to an 8.4-fold increase in titer and demonstrates the utility of the sesquiterpene platform strain.

### Triterpene Platform Strain

**FIGURE 4:**
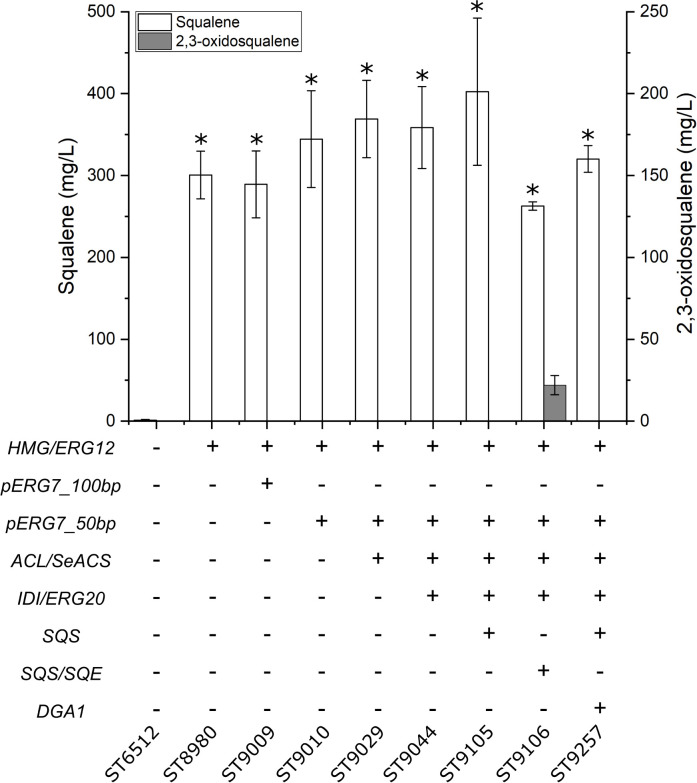
Squalene and 2,3-oxidosqualene production by the triterpene platform strain. HMG, HMG-reductase; *ERG12*, mevalonate kinase; *pERG7_100bp* and *pERG7_50bp*, truncation of the native lanosterol synthase promoter to 100 or 50 bp, respectively, immediately upstream of the *ERG7*-gene; *ACL*, ATP citrate lyase 1; *SeACS*, *S. enterica* acetyl-CoA synthetase; *IDI*, isopentyl diphosphate isomerase; *ERG20*, farnesyl diphosphate synthase; *SQS*, squalene synthase; *SQE*, squalene epoxidase; *DGA1*, diacylglycerol O-acyltransferase 1. Standard deviations from the values of three biological replicates are represented by error bars. Asterisk marks statistical significant changes compared to ST6512 (student’s *t*-test, two-tailed, *p* < 0.05).

Squalene is a sought-after compound as a food and cosmetics additive, while many complex triterpenoids have pharmaceutical properties (Xu et al., 2016; Hill and Connolly, 2017). Therefore, platform strains for the production of squalene and complex triterpenoids were constructed. This was achieved by consecutively modifying the base strain (ST6512) through several rounds of engineering. Since squalene is naturally produced by *Y. lipolytica*, we could test the production in each intermediate strain constructed toward the final squalene and triterpene platform strain. First, *HMG* and *ERG12* were expressed which resulted in the production of 300.7 ± 29.1 mg/L squalene (Figure 4). Secondly, the native lanosterol promoter was truncated to leave either 100 bp or 50 bp of the promoter directly upstream of the lanosterol synthase gene (*pERG7_100bp* or *pERG7_50bp*, respectively) generating ST9009 and ST9010, respectively. The lanosterol synthase catalyzes the committed step toward sterols from 2,3-oxidosqualene and limiting this step could potentially increase the pool of 2,3-oxidosqualene and squalene. Building on ST9010, *ACL* and *SeACS* were expressed and subsequently, *IDI* and *ERG20* were overexpressed. Further overexpression of the native squalene synthase (*SQS*) resulted in squalene platform strain which produced 402.4 ± 90 mg/L squalene. When instead *SQE* and *SQS* were co-overexpressed generating the complex triterpenoid platform strain (ST9106), only 262.7 ± 5.2 mg/L squalene were produced, while levels of 22 ± 5.9 mg/L 2,3-oxidosqualene was detected. Indeed, 2,3-oxidosqualene was measured for all the squalene producing strains but could only be detected in the complex triterpenoid platform strain. Overexpression of the native diacylglycerol O-acyltransferase (*DGA1*) together in the squalene platform strain resulted in 320 ± 16.2 mg/L squalene, less than when only expressing *SQS*. Overexpression of the native *DGA1* in combination with a truncated version of *HMG* in *S. cerevisiae* increased squalene titers, probably due to an increased lipid content providing storage for squalene accumulation (Wei et al., 2018). However, overexpression of *DGA1* in *Y. lipolytica* did not benefit squalene production in the context of this study, likely due to the diversion of acetyl-CoA to lipid biogenesis (Figure 1).

### Diterpene and Carotenoid Platform Strain

**FIGURE 5:**
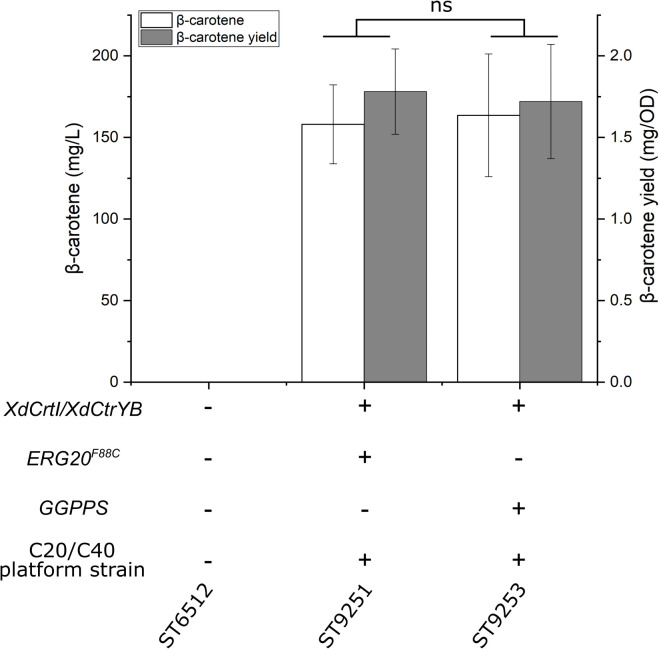
β-carotene production by the diterpene/carotenoid platform strains. *XdCrtI*, *X. dendrorhous phytoene desaturase*; *XdCrtYB*, *X. dendrorhous* bi-functional phytoene synthase/lycopene cyclase; *ERG20^*F*88*C*^*, mutated version with geranylgeranyl diphosphate synthase activity; *GGPPS*, geranylgeranyl diphosphate synthase. The diterpene/carotenoid (C20/C40) platform strain contains the following gene overexpressions *HMG*, *ERG12*, *ACL*, *SeACS*, and *IDI*, while the native squalene promoter was swapped (*pERG11_SQS*). Average values and standard deviations for each strain are based on two individual clones with three biological replicates each. Statistical non-significance is marked with ns (student’s *t*-test, two-tailed, *p* > 0.05).

To engineer a platform strain for the production of diterpenoids and carotenoids, the genes *HMG*, *ERG12*, *ACL*, *SeACS*, and *IDI* were overexpressed, while the native squalene promoter was swapped with the relatively weak promoter *pERG11* to reduce the flux toward squalene (*pERG11_SQS*). Additionally, either *GGPPS* or *ERG20^*F*88*C*^* was overexpressed to generate the diterpenoid/carotenoid platform strains ST9150 and ST9203, respectively. To test the potential of these platform strains, the genes encoding phytoene desaturase (*XdCtrl*) and bi-functional phytoene synthase/lycopene cyclase (*XdCtrYB*) from *X. dendrorhous* for the production of β-carotene were expressed in both strains, generating ST9251 and ST9253, respectively. ST9251 produced 158 ± 24.1 mg/L β-carotene, while ST9253 produced 164 ± 37.6 mg/L β-carotene (Figure 5).

## Discussion

This study displays the capability of the oleaginous yeast *Y. lipolytica* for terpenoid production by the construction of tailored terpene platform strains. Each platform strain was designed for increased production of either mono-, sesqui-, tri-, or diterpenoids and carotenoids, and production potential was tested with terpenoids from the appropriate class. The effects of four different metabolic engineering strategies for sesquiterpenoid production were investigated in a strain expressing *AaBFS* for β-farnesene. Overexpression of *HMG* and *ERG12* had previously been shown to increase limonene production 112-fold to 23.56 mg/L in *Y. lipolytica* (Cao et al., 2016). The strategy of *HMG*-overexpression is very commonly used for terpenoid production in *Y. lipolytica*, with both truncated and non-truncated versions, and variants from other species being utilized, while the *ERG12* gene often is overexpressed together with the whole MVA-pathway (Table 1). Indeed, overexpression *ERG12* and *HMG* increased β-farnesene production approximately 3-fold, from 212.7 ± 5.6 mg/L to 631 ± 46.1 mg/L, demonstrating that this strategy works well for β-farnesene production. By further overexpression of *ERG20*, *IDI*, *SeACS*, and *ACL1* the titer of 955 ± 45.1 mg/L β-farnesene was reached. Improvement of acetyl-CoA by overexpression of *SeACS* and *ACL1* had a favorable effect on β-farnesene production which is consistent with previous reports demonstrating a similar effect for squalene production in *Y. lipolytica* (Huang et al., 2018). However, recent studies have found that strategies that increase β-oxidation such as overexpression of multifunctional β-oxidation enzyme 1 (*MFE1*) or implementing alternative routes for acetyl-CoA biogenesis by expression of phosphoketolases (*PK*) and phosphotransacetylases (*PTA*), the non-glycolytic oxidation pathway, can also increase terpenoid titers (Table 1; Jin et al., 2019; Lu et al., 2020). It would be highly interesting for future research to compare these strategies for terpenoid production in a systematic manner. β-farnesene has been produced at titers of 130 g/L by highly modified *S. cerevisiae* in 200,000-liter bioreactors and therefore is an excellent terpene to demonstrate the effect of metabolic engineering strategies (Meadows et al., 2016). While β-farnesene has not been produced previously in *Y. lipolytica*, the isomer α-farnesene has been produced by engineered *Y. lipolytica* at 25.55 g/L in 1-liter bioreactors (Liu et al., 2019). Interestingly, switching the endogenous squalene promoter with the weak *pERG11* promoter did not benefit β-farnesene production, although the same strategy was shown to boost β-carotene production in *Y. lipolytica* (Kildegaard et al., 2017). This could be caused the combination of reduced cellular sterol-content and high β-farnesene levels that may alter the lipid membrane properties and induce toxicity. It has been demonstrated that several low-molecular-weight terpenoids like limonene, geraniol, and pinene can be toxic to *S. cerevisiae* (Uribe et al., 1985; Liu et al., 2013; Zhao et al., 2016). Indeed, pinene was shown to potentially increase lipid membrane fluidity, while limonene could adversely affect the yeast cell wall (Brennan et al., 2013). *Candida albicans* cells exposed to high doses of linalool or its derivative linalyl acetate displayed apoptotic phenotypes and highly fluidized membranes leading to cell death (Khan et al., 2014; Blaskó et al., 2017). Two-phase cultivation with an organic phase has been shown to alleviate the toxicity of small terpenoids against *S. cerevisiae* (Brennan et al., 2012). Indeed, a dodecane phase can be used to reduce cell toxicity and to accumulate the produced compounds (Tippmann et al., 2016). It is therefore likely that the utilization of a dodecane phase for the accumulation of limonene, valencene, and β-farnesene also helped to limit adverse effects in *Y. lipolytica* in this study. The sesquiterpene valencene is the precursor of nootkatone, a compound used for cosmetics and fragrances, with insect repellant properties (Leonhardt and Berger, 2014). By simply integrating *CnVS* in the optimized sesquiterpene platform strain, 113.9 ± 6.6 mg/L valencene was produced, an approximately 8.4-fold increase over the base strain. This example clearly illustrates the usefulness of pre-engineered strains for achieving high terpene titers quickly and without post-optimization of the strain. By comparison, previous valence production as precursor for nootkatone in engineered *Y. lipolytica* resulted in 22.8 mg/L valencene and 978.2 μg/L nootkatone during shake flask cultivation (Guo et al., 2018). The sesquiterpene platform strain is a powerful platform for the production of terpenes like valencene and could be engineered to produce more complex sesquiterpenoids. Limonene has been produced at 23.56 mg/L and 1.36 mg/gDCW in shake flasks by the *Y. lipolytica* strain Pof1-LN-051 expressing a limonene synthase from Korean mint *Agastache rugosa* (Cao et al., 2016). In a subsequent report, an additional limonene synthase copy was expressed in Pof1-LN-051, where after cultivation in 1.5 L bioreactors resulted in 165.3 mg/L, which is the highest reported limonene titer for *Y. lipolytica* (Cheng et al., 2019). By comparison, the expression of *PfLS* in the monoterpenoid platform strain resulted in 35.9 ± 1.1 mg/L and 1.46 ± 0.064 mg/gDCW limonene in small-scale cultivation. Therefore, the monoterpene platform strain clearly holds a great potential for limonene production, which potentially could be improved substantially by multi-copy integration of *PfLS* and scale-up into bioreactors. By integrating *HMG*, *ERG12*, *ERG20*, *IDI, SQS*, and truncating the native lanosterol promoter, a strain for squalene production was constructed. This strain was able to produce 402.4 ± 90 mg/L squalene by small-scale cultivation. Other reports of squalene production in highly modified *Y. lipolytica* show titers up to 531.6 mg/L by a strain with at least twelve modifications relevant to squalene production, including overexpression of the entire MVA-pathway and knockouts of peroxisomal β-oxidation (*pox*) genes which could affect acetyl-CoA balance (Table 1; Gao et al., 2017). By comparison the squalene platform strain from this study contained only seven gene overexpression and one gene downregulation. Therefore, further engineering and scale-up could potentially improve the production of squalene described herein. Overexpression of *SQE* in combination with the modifications described for the squalene production strain resulted in 22 ± 5.9 mg/L 2,3-oxidosqualene. This platform strain can potentially be used for the production of complex triterpenoids. Since *SQE* was expressed under the *pGPD*-promoter, expressing additional copies of *SQE*, potentially under the relatively stronger *pTEFintron*-promoter, could raise 2,3-oxidosqualene levels further (Holkenbrink et al., 2018). The production of β-carotene for the carotenoid platform strains, 158 ± 24.1 mg/L or 164 ± 37.6 mg/L for ST9251 or ST9253, respectively, are lower than in previous reports (Kildegaard et al., 2017; Larroude et al., 2018). To achieve 6.5 g/L β-carotene, three copies of the pathway enzymes (*carB* and *CarRP*) and *GGPPS* were integrated in a W29-derived strain engineered for high lipogenesis (Larroude et al., 2018). Therefore, further engineering of the downstream biosynthetic pathway may be necessary when using the platform strains.

## Conclusion

Tailored platform strains for the production of mono-, sesqui-, tri-, or diterpenes and carotenoids were constructed, and their potential was tested by the production of limonene, β-farnesene and valencene, squalene and 2,3-oxidosqualene, and β-carotene, respectively. These improved platform strains were metabolically engineered for improved terpenoid production and their use could result in almost 100-fold improvement production for some terpenes compared to base strains. These platform strains can potentially be improved even further by engineering and scale-up into bioreactors.

## Strain Availability Statement

The platform strains and representative terpenoid producing strains from this study can be requested from Euroscarf. The Euroscarf accession numbers are given in parenthesis. ST9202, monoterpene platform strain (Y41400). ST9250, monoterpene platform strain with *PfLS* (Y41401). ST9395, base strain with *PfLS* (Y41402). ST9149, sesquiterpene platform strain (Y41403). ST9204, sesquiterpene platform strain with *CnVS* (Y41404). ST9396, base strain with *CnVS* (Y41405). ST9105, squalene production strain (Y41406). ST9106, triterpene platform strain (Y41407). ST6512, base strain (Y41408). ST9203, diterpene/carotenoid platform strain with *ERG20*^*F88C*^ (Y41409). ST9150, diterpene/carotenoid platform strain with GGPPS (Y41410). The β-farnesene test-strains ST8979, ST8998, ST9006, ST9026, and ST9147 (Y41411-15).

## Data Availability Statement

The platform strains and representative strains for terpenoid production can be requested from Euroscarf or the authors.

## Author Contributions

JA, KK, and IB designed the experiments. MC, SJ, and JA carried out the experiments. MK provided critical assistance in developing GC-FID and HP-LC methods. JA analyzed the data. JA and IB wrote the manuscript. All authors contributed to the article and approved the submitted version.

## Conflict of Interest

The authors declare that the research was conducted in the absence of any commercial or financial relationships that could be construed as a potential conflict of interest.
