# Supplementary material for: Yarrowia lipolytica Strains Engineered for the Production of Terpenoids
Source: Front Bioeng Biotechnol. 2020 Aug 14;8:945. doi: 10.3389/fbioe.2020.00945 (PMC7456906; doi:10.3389/fbioe.2020.00945)
Supplement: Supplementary file 1 [file Data_Sheet_1.pdf]

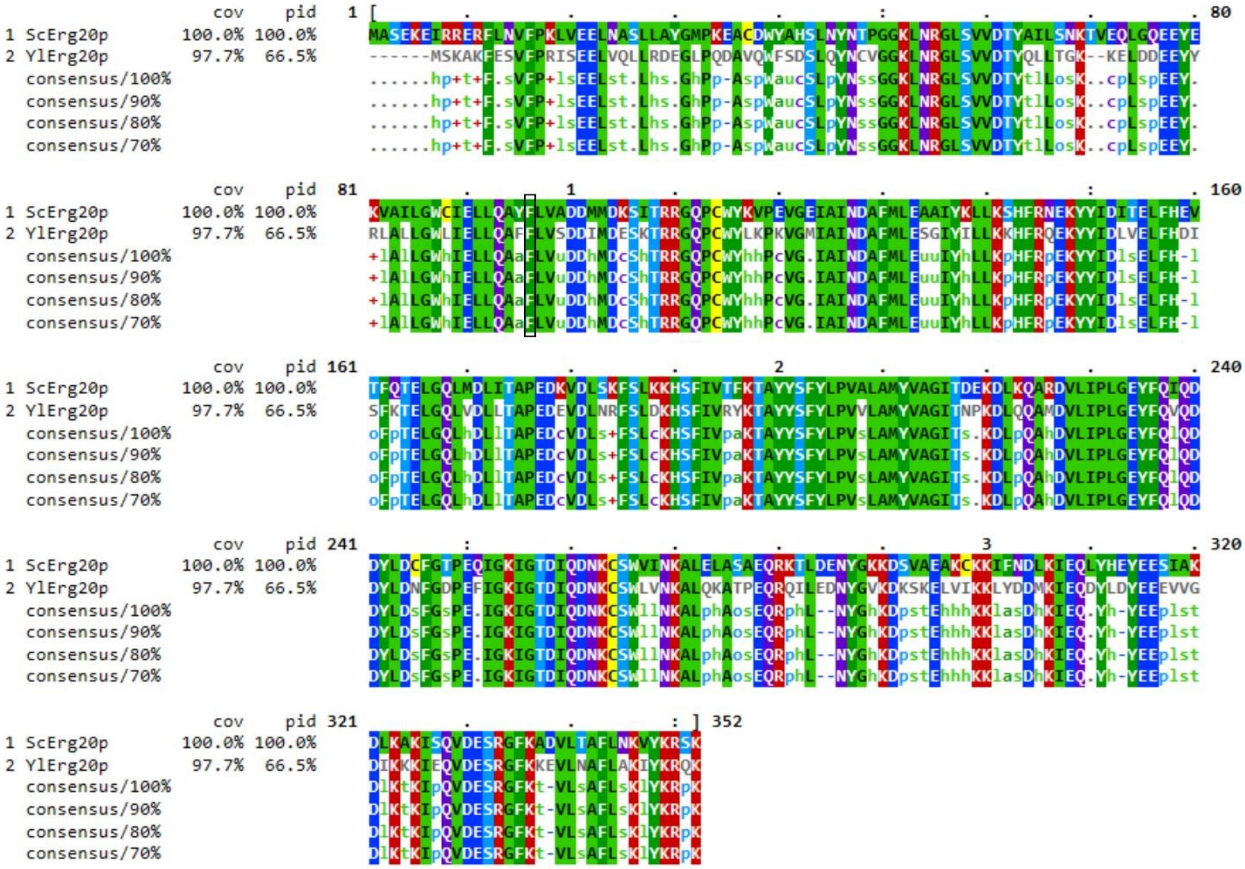

Figure 1: Alignment of *Y. lipolytica* Erg20p (YlERG20p. Accession number: XP_503599.1) and *S. cerevisiae* Erg20p (ScERG20p. Accession number: CAA89462.1) amino sequences. The black box marks the phenylalanine residue in position 96 of ScERG20p and 88 of YlERG20p that was substituted for cysteine in the mutated version *Y. lipolytica* Erg20p^{F88C}.

Supplementary table 1. Strains used in this study.

Strain ID	Genotypes	Parent strain	gRNA-vector	integration vector/biobrick	Reference
ST3683	*mus51*Δ, *nugm*-Htg2, *ndh2*i, *lys11*-, *leu2*-, *ura3*-, MatB				(Angerer et al., 2014) *Y. lipolytica* GB20 was a kind gift from Volker Zickermann.
ST4842	MATa				*Y. lipolytica* W29 (MatA, ATCC©20460TM) strain Y-63746 from the ARS culture collection
ST6512	MATa ku70Δ::PrTEF1->Cas9-TTef12::PrGPD->DsdA-TLip2	ST4842		pCfB6364	(Marella et al., 2019)
		**β-farnesene test strain**			
ST8979	MATa ku70Δ::PrTEF1->Cas9-TTef12::PrGPD->DsdA-TLip2 IntE_1-PrTefInt->AaBFS	ST6512	pCfB6633	pCfB8829	This study
ST8998	MATa ku70Δ::PrTEF1->Cas9-TTef12::PrGPD->DsdA-TLip2	ST8979	pCfB8855	pCfB8822	This study

	IntE_1-PrTefInt->AaBFS IntC_2-HMG1<-PrGPD-PrTefInt->ERG12				
ST9006	MATa ku70Δ::PrTEF1->Cas9-TTef12::PrGPD->DsdA-TLip2 IntE_1-PrTefInt->AaBFS IntC_2-HMG1<-PrGPD-PrTefInt->ERG12 IntD_1-IDI1<-PrGPD-PrTefInt->ERG20	ST8998	pCfB6631	pCfB8878	This study
ST9026	MATa ku70Δ::PrTEF1->Cas9-TTef12::PrGPD->DsdA-TLip2 IntE_1-PrTefInt->AaBFS IntC_2-HMG1<-PrGPD-PrTefInt->ERG12 IntD_1-IDI1<-PrGPD-PrTefInt->ERG20 IntC_3-SeACS<-PrGPD-PrTefInt->YlACL1	ST9006	pCfB8856	pCfB8823	This study
ST9147	MATa ku70Δ::PrTEF1->Cas9-TTef12::PrGPD->DsdA-TLip2 IntE_1-PrTefInt->AaBFS IntC_2-HMG1<-PrGPD-PrTefInt->ERG12 IntD_1-IDI1<-PrGPD-PrTefInt->ERG20 IntC_3-SeACS<-PrGPD-PrTefInt->YlACL1 pERG11::pSQS1	ST9026	pCfB8978	BB4010	This study
	**Sesquiterpene platform strain**				
ST8980	MATa ku70Δ::PrTEF1->Cas9-TTef12::PrGPD->DsdA-TLip2 IntC_2-HMG1<-PrGPD-PrTefInt->ERG12	ST6512	pCfB6627	pCfB8822	This study
ST9027	MATa ku70Δ::PrTEF1->Cas9-TTef12::PrGPD->DsdA-TLip2 IntC_2-HMG1<-PrGPD-PrTefInt->ERG12 IntC_3-SeACS<-PrGPD-PrTefInt->YlACL1	ST8980	pCfB8856	pCfB8823	This study
ST9100	MATa ku70Δ::PrTEF1->Cas9-TTef12::PrGPD->DsdA-TLip2 IntC_2-HMG1<-PrGPD-PrTefInt->ERG12 IntC_3-SeACS<-PrGPD-PrTefInt->YlACL1 IntD_1-IDI1<-PrGPD-PrTefInt->ERG20	ST9027	pCfB6631	pCfB8878	This study
ST9149	MATa ku70Δ::PrTEF1->Cas9-TTef12::PrGPD->DsdA-TLip2 IntC_2-HMG1<-PrGPD-PrTefInt->ERG12 IntC_3-SeACS<-PrGPD-PrTefInt->YlACL1 IntD_1-IDI1<-PrGPD-PrTefInt->ERG20 pERG11::pSQS1	ST9100	pCfB8979	BB4010	This study
ST9204	MATa ku70Δ::PrTEF1->Cas9-TTef12::PrGPD->DsdA-TLip2 IntC_2-HMG1<-PrGPD-PrTefInt->ERG12 IntC_3-SeACS<-PrGPD-PrTefInt->YlACL1 IntD_1-IDI1<-PrGPD-PrTefInt->ERG20 pERG11::pSQS1 IntE_1-PrTefInt->CnVS	ST9149	pCfB6633	pCfB8830	This study
ST9396	MATa ku70Δ::PrTEF1->Cas9-TTef12::PrGPD->DsdA-TLip2 IntE_1-PrTefInt->CnVS	ST6512	pCfB6633	pCfB8830	This study
	**Monoterpene platform strain**				
ST9148	MATa ku70Δ::PrTEF1->Cas9-TTef12::PrGPD->DsdA-TLip2 IntC_2-HMG1<-PrGPD-PrTefInt-	ST9027	pCfB8978	BB4010	This study

	>ERG12 IntC_3-SeACS<-PrGPD-PrTefInt->YlACL1 pERG11::pSQS1				
ST9202	MATa ku70Δ::PrTEF1->Cas9-TTef12::PrGPD->DsdA-TLip2 IntC_2-HMG1<-PrGPD-PrTefInt->ERG12 IntC_3-SeACS<-PrGPD-PrTefInt->YlACL1 pERG11::pSQS1 IntD_1-IDI1<-PrGPD-PrTefInt->ERG20(F88W-N119W)	ST9148	pCfB8857	pCfB8824	This study
ST9249	MATa ku70Δ::PrTEF1->Cas9-TTef12::PrGPD->DsdA-TLip2 IntC_2-HMG1<-PrGPD-PrTefInt->ERG12 IntC_3-SeACS<-PrGPD-PrTefInt->YlACL1 pERG11::pSQS1 IntD_1-IDI1<-PrGPD-PrTefInt->ERG20(F88W-N119W) IntE_1-PrTefInt->ClLS	ST9202	pCfB6633	pCfB8827	This study
ST9250	MATa ku70Δ::PrTEF1->Cas9-TTef12::PrGPD->DsdA-TLip2 IntC_2-HMG1<-PrGPD-PrTefInt->ERG12 IntC_3-SeACS<-PrGPD-PrTefInt->YlACL1 pERG11::pSQS1 IntD_1-IDI1<-PrGPD-PrTefInt->ERG20(F88W-N119W) IntE_1-PrTefInt->PfLS	ST9202	pCfB6633	pCfB8828	This study
ST9394	MATa ku70Δ::PrTEF1->Cas9-TTef12::PrGPD->DsdA-TLip2 IntE_1-PrTefInt->ClLS	ST6512	pCfB6633	pCfB8827	This study
ST9395	MATa ku70Δ::PrTEF1->Cas9-TTef12::PrGPD->DsdA-TLip2 IntE_1-PrTefInt->PfLS	ST6512	pCfB6633	pCfB8828	This study
**Triterpene platform strain**					
ST9009	MATa ku70Δ::PrTEF1->Cas9-TTef12::PrGPD->DsdA-TLip2 IntC_2-HMG1<-PrGPD-PrTefInt->ERG12 pERG7_100bp	ST8980	pCfB8880	BB3895	This study
ST9010	MATa ku70Δ::PrTEF1->Cas9-TTef12::PrGPD->DsdA-TLip2 IntC_2-HMG1<-PrGPD-PrTefInt->ERG12 pERG7_50bp	ST8980	pCfB8880	BB3952	This study
ST9029	MATa ku70Δ::PrTEF1->Cas9-TTef12::PrGPD->DsdA-TLip2 IntC_2-HMG1<-PrGPD-PrTefInt->ERG12 pERG7_50bp IntC_3-SeACS<-PrGPD-PrTefInt->YlACL1	ST9010	pCfB6630	pCfB8823	This study
ST9044	MATa ku70Δ::PrTEF1->Cas9-TTef12::PrGPD->DsdA-TLip2 IntC_2-HMG1<-PrGPD-PrTefInt->ERG12 pERG7_50bp IntC_3-SeACS<-PrGPD-PrTefInt->YlACL1 IntD_1-IDI1<-PrGPD-PrTefInt->ERG20	ST9029	pCfB8857	pCfB8878	This study
ST9105	MATa ku70Δ::PrTEF1->Cas9-TTef12::PrGPD->DsdA-TLip2 IntC_2-HMG1<-PrGPD-PrTefInt->ERG12 pERG7_50bp IntC_3-SeACS<-PrGPD-PrTefInt->YlACL1 IntD_1-IDI1<-PrGPD-PrTefInt->ERG20 IntE_1-PrTefInt->SQS1	ST9044	pCfB6633	pCfB8832	This study
ST9106	MATa ku70Δ::PrTEF1->Cas9-TTef12::PrGPD->DsdA-TLip2	ST9044	pCfB6633	pCfB8831	This study

	IntC_2-HMG1<-PrGPD-PrTefInt->ERG12 pERG7_50bp IntC_3-SeACS<-PrGPD-PrTefInt->YlACL1 IntD_1-IDI1<-PrGPD-PrTefInt->ERG20 IntE_1-SQE<-PrGPD-PrTefInt->SQS1				
ST9257	MATa ku70Δ::PrTEF1->Cas9-TTef12::PrGPD->DsdA-TLip2 IntC_2-HMG1<-PrGPD-PrTefInt->ERG12 pERG7_50bp IntC_3-SeACS<-PrGPD-PrTefInt->YlACL1 IntD_1-IDI1<-PrGPD-PrTefInt->ERG20 IntE_1-PrTefInt->SQS1 IntE_3-PrTefInt->DGA1	ST9105	pCfB8860	pCfB8837	This study
**Diterpene and carotenoid platform strains**					
ST9102	MATa ku70Δ::PrTEF1->Cas9-TTef12::PrGPD->DsdA-TLip2 IntC_2-HMG1<-PrGPD-PrTefInt->ERG12 IntC_3-SeACS<-PrGPD-PrTefInt->YlACL1 IntD_1-IDI1<-PrGPD-PrTefInt->GGS1	ST9027	pCfB6631	pCfB8826	This study
ST9148	MATa ku70Δ::PrTEF1->Cas9-TTef12::PrGPD->DsdA-TLip2 IntC_2-HMG1<-PrGPD-PrTefInt->ERG12 IntC_3-SeACS<-PrGPD-PrTefInt->YlACL1 pERG11::pSQS1	ST9027	pCfB8978	BB4010	This study
ST9150	MATa ku70Δ::PrTEF1->Cas9-TTef12::PrGPD->DsdA-TLip2 IntC_2-HMG1<-PrGPD-PrTefInt->ERG12 IntC_3-SeACS<-PrGPD-PrTefInt->YlACL1 IntD_1-IDI1<-PrGPD-PrTefInt->GGS1 pERG11::pSQS1	ST9102	pCfB8979	BB4010	This study
ST9203	MATa ku70Δ::PrTEF1->Cas9-TTef12::PrGPD->DsdA-TLip2 IntC_2-HMG1<-PrGPD-PrTefInt->ERG12 IntC_3-SeACS<-PrGPD-PrTefInt->YlACL1 pERG11::pSQS1 IntD_1-IDI1<-PrGPD-PrTefInt->ERG20(F96C)	ST9148	pCfB8857	pCfB8825	This study
ST9251	MATa ku70Δ::PrTEF1->Cas9-TTef12::PrGPD->DsdA-TLip2 IntC_2-HMG1<-PrGPD-PrTefInt->ERG12 IntC_3-SeACS<-PrGPD-PrTefInt->YlACL1 pERG11::pSQS1 IntD_1-IDI1<-PrGPD-PrTefInt->ERG20(F96C) IntE_1-XdCrtl<-PrGPD-PrTefInt->XdCtrYB	ST9203	pCfB6633	pCfB8833	This study
ST9253	MATa ku70Δ::PrTEF1->Cas9-TTef12::PrGPD->DsdA-TLip2 IntC_2-HMG1<-PrGPD-PrTefInt->ERG12 IntC_3-SeACS<-PrGPD-PrTefInt->YlACL1 IntD_1-IDI1<-PrGPD-PrTefInt->GGS1 pERG11::pSQS1 IntE_1-XdCrtl<-PrGPD-PrTefInt->XdCtrYB	ST9150	pCfB6633	pCfB8833	This study

Supplementary table 2. Vectors used in this study.

Vector Name	Parent vector	Biobricks	References
pBP8009 (pIntF_3-TPex20-TLip2)			(Holkenbrink et al., 2018)

pCfB3405 (pORI1001-Nat-CEN1-USER)			(Holkenbrink et al. 2018)
pCfB5119 (pIntB-HphMx-YlHMG1<-PrGPD-PrFBA1->YlGGS1)			(Kildegaard et al., 2017)
pCfB5141 (pIntE-1-NatMx-XdCrtI<-PrGPD-PrFBA1->XdCrtYB)			(Kildegaard et al., 2017)
pCfB6364 (ku70Δ::SpCas9-EcDsdAMX4)			(Marella et al., 2019)
pCfB6371 (pIntC_3-TPex20-TLip2)			(Holkenbrink et al. 2018)
pCfB6601 (pIntE-4-Hph-PrExp->PfLS)			Unpublished
pCfB6602 (pIntE-4-Hph-PrExp->ClLS)			Unpublished
pCfB6605 (pIntE-4-Hph-PrExp->YlSQS1)			Unpublished
pCfB6606 (pIntE-4-Hph-CnVS<-PrEXP)			Unpublished
pCfB6620 (pORI1001-Nat-CEN1-USER-IDI1<-PrEXP-PrGPD->ERG20)			Unpublished
pCfB6677 (pIntE_1-TPex20-TLip2)			(Holkenbrink et al. 2018)
pCfB6679 (pIntE_4-TPex20-TLip2)			(Holkenbrink et al. 2018)
pCfB6681 (pIntE_3-TPex20-TLip2)			(Holkenbrink et al. 2018)
pCfB6682 (pIntC_2-TPex20-TLip2)			(Holkenbrink et al. 2018)
pCfB6684 (pIntD_1-TPex20-TLip2)			(Holkenbrink et al. 2018)
pCfB6799 (pIntE-4-Hph-BFS_M13<-PrEXP)			Unpublished
pCfB7063 (prDNA-Ura3d1-TPex20+PTEfintron+CrtW+TLip2)			(Kildegaard et al., 2017)
pCFB8843 (pORI1001-Hyg-CEN1-USER)		BB3924, BB3925	This study
**Integration vectors**			
pCfB8822 (IntC_2-HMG1<-PrGPD-PrTefInt->ERG12)	pCfB6682	BB3865, BB3866, BB3867	This study
pCfB8823 (IntC_3-SeACS<-PrGPD-PrTefInt->YlACL1)	pCfB6371	BB3865, BB3868, BB3869	This study
pCfB8824 (IntD_1-IDI1<-PrGPD-PrTefInt->ERG20(F88W-N119W))	pCfB6684	BB3865, BB3870, BB3874	This study
pCfB8825 (IntD_1-IDI1<-PrGPD-PrTefInt->ERG20(F96C))	pCfB6684	BB3865, BB3870, BB3877	This study
pCfB8826 (IntD_1-IDI1<-PrGPD-PrTefInt->GGS1)	pCfB6684	BB3865, BB3870, BB3878	This study
pCfB8827 (IntE_1-PrTefInt->ClLS)	pCfB6677	BB3879, BB3880	This study
pCfB8828 (IntE_1-PrTefInt->PfLS)	pCfB6677	BB3879, BB3881	This study
pCfB8829 (IntE_1-PrTefInt->AaBFS)	pCfB6677	BB3879, BB3882	This study
pCfB8830 (IntE_1-PrTefInt->CnVS)	pCfB6677	BB3879, BB3883	This study
pCfB8831 (IntE_1-SQE<-PrGPD-PrTefInt->SQS1)	pCfB6677	BB3865, BB3884, BB3885	This study
pCfB8832 (IntE_1-PrTefInt->SQS1)	pCfB6677	BB3879, BB3885	This study
pCfB8833 (IntE_1-XdCrtl<-PrGPD-PrTefInt->XdCtrYB)	pCfB6677	BB3865, BB3886, BB3887	This study
pCfB8837 (IntE_3-PrTefInt->DGA1)	pCfB6681	BB3879, BB3898	This study
pCfB8878 (IntD_1-IDI1<-PrGPD-PrTefInt->ERG20)	pCfB6684	BB3865, BB3870, BB3871	This study
**gRNA-vectors**			
pCfB6627 (pNat-YLgRNA2_IntC_2)			(Holkenbrink et al. 2018)
pCfB6630 (pNat-YLgRNA3_IntC_3)			(Holkenbrink et al. 2018)
pCfB6631 (pNat-YLgRNA2_IntD_1)			(Holkenbrink et al. 2018)
pCfB6633 (pNat-YLgRNA2_IntE_1)			(Holkenbrink et al. 2018)

pCfB6637 (pNat-YLgRNA3_IntE_3)			(Holkenbrink et al. 2018)
pCfB6638 (pNat-YLgRNA2_IntE_4):			(Holkenbrink et al. 2018)
pCfB8855 (pHphM-YLgRNA2_IntC_2)	pCFB8843	BB3926	This study
pCfB8856 (pHphM-YLgRNA2_IntC_3)	pCFB8843	BB3927	This study
pCfB8857 (pHphM-YLgRNA2_IntD_1)	pCFB8843	BB3928	This study
pCfB8858 (pHphM-YLgRNA2_IntE_1)	pCFB8843	BB3929	This study
pCfB8880 (pERG7_gRNA_KO_Hyg)	pCFB8843	BB1635, BB1636, PR-24059, PR-24060	This study
pCfB8978 (pSQS1_replacement_gRNA_Nat)	pCfB3405	BB1635, BB1636, PR-24587, PR-24588	This study
pCfB8979 (pSQS1_replacement_gRNA_Hyg)	pCFB8843	BB1635, BB1636, PR-24587, PR-24588	This study
**Synthetic genes**			
pCfB8820 (SeACS)			This study

Supplementary table 3. Biobricks used in this study.

**Biobrick Name**	**Template**	**Forward primer**	**Reverse primer**	**Reference**
BB1635				(Holkenbrink et al. 2018)
BB1636				(Holkenbrink et al. 2018)
BB3863 (Tefint(PrGDPfusion)->)	pCfB7063	24013	18214	This study
BB3864 (<-PrGDP)	pCfB5119	15528	15529	This study
BB3865 (<-PrGDP_Tefint->)	BB3863, BB3864	15528	18214	This study
BB3866 (<-HMG1)	pCfB5119	23753	23752	This study
BB3867 (ERG12->)	ST3683 gDNA	24014	24015	This study
BB3868 (<-YlOpSeACS)	pCfB8820	24016	24017	This study
BB3869 (YlACL1->)	ST3683 gDNA	24018	24019	This study
BB3870 (<-IDI1)	ST3683 gDNA	24020	24021	This study
BB3871 (ERG20->)	pCfB6620	24022	24023	This study
BB3871 (ERG20->)	pCfB6620	24022	24023	This study
BB3872 (ERG20(F88W-N119W)_up)	pCfB6620	24022	24026	This study
BB3873 (ERG20(F88W-N119W)_down)	pCfB6620	24027	24023	This study
BB3874 (ERG20(F88W-N119W)->)	BB3872, BB3873	24022	24023	This study
BB3875 (ERG20(F88C)_up)	pCfB6620	24022	24024	This study
BB3876 (ERG20(F88C)_down)	pCfB6620	24025	24023	This study
BB3877 (ERG20(F88C)->)	BB3875, BB3876	24022	24023	This study
BB3878 (GGS1->)	pCfB5119	24028	24029	This study
BB3879 (Tefint->)	pCfB7063	23847	18214	This study
BB3880 (ClLS->)	pCfB6602	24030	24031	This study
BB3881 (PfLS->)	pCfB6601	24032	24033	This study
BB3882 (AaBFS->)	pCfB6799	24061	24062	This study
BB3883 (CnVS->)	pCfB6606	24034	24035	This study
BB3884 (<-SQE)	ST3683 gDNA	24038	24039	This study
BB3885 (SQS1->)	pCfB6605	24036	24037	This study

BB3886 (<-XdCrtl)	pCfB5141	24044	24045	This study
BB3887 (XdCtrYB->)	pCfB5141	24046	24047	This study
BB3890 (pSQS_50bp_up)	ST3683 gDNA	24048	24049	This study
BB3891 (pSQS_50bp_down)	ST3683 gDNA	17760	24050	This study
BB3893 (pERG7_100bp_up)	ST3683 gDNA	24055	24056	This study
BB3894 (pERG7_100bp_down)	ST3683 gDNA	24057	24058	This study
BB3895 (pERG7_100bp_repair)	BB3893, BB3894	24055	24058	This study
BB3898 (DGA1_YALI0E32769g->)	ST3683 gDNA	24365	24070	This study
BB3924 (Episomal vector backbone w/o HphMX)	pCFB3405	23934	10593	This study
BB3925 (HphMX-TTef1 insert)	pCfB6605	23935	23936	This study
BB3926 (gRNA-cassette for IntC_2)	pCFB6627	10607	10604	This study
BB3927 (gRNA-cassette for IntC_3)	pCfB6630	10607	10604	This study
BB3928 (gRNA-cassette for IntD_1)	pCfB6631	10607	10604	This study
BB3929 (gRNA-cassette for IntE_1)	pCfB6633	10607	10604	This study
BB3944 (pERG7_50bp_up)	ST3683 gDNA	24055	24366	This study
BB3952 (pERG7_50bp_repair)	BB3944, BB3894	24055	24058	This study
BB4007 (SQS_UP_promoter_replacement)	ST3683 gDNA	24581	24582	This study
BB4008 (pERG11)	ST3683 gDNA	24583	24584	This study
BB4009 (SQS_DOWN_promoter_replacement)	ST3683 gDNA	24585	24586	This study
BB4010 (SQS_UP_pERG11_pSQS_DOWN)	BB4007, BB4008, BB4009	24581	24586	This study

Supplementary table 4. Primers used in this study.

**Primer Name**	**Sequence (5'->3')**
PR-10593	AGCAGGCTUGGAGGCGACGTGGCAG
PR-10604	CACGCGAUACCGTACCCACACAAAAAAAGCACCACCGACTC
PR-10607	CGTGCGAUAGTGAATCATTGCTAACAGATC
PR-15528	ATGACAGAUTGTTGATGTGTGTTTAATTCAAGAATG
PR-15529	AGCTACTGAUGACGCAGTAGGATGTCCTGCACGG
PR-17760	AGTGGCCUATTAGCAACACGATCTACC
PR-18214	AGTACTGCAAAAAGUGCTG
PR-18214	AGTACTGCAAAAAGUGCTG
PR-23752	ATCTGTCAUGCCACAATGCTACAAGCAGCTATTGG
PR-23753	CACGCGAUCTATGACCGTATGCAAATATTCG
PR-23847	CGTGCGAUAGAGACCGGGTTGGCGGCGCAT
PR-23934	ACCCATTGCTGUAGATATGTCTTGTGTGTAAGGGGG
PR-23935	ACAGCAATGGGUAAAAAGCCTGAACTCACCGC
PR-23936	AAGCCTGCUGAATTCGGACACGGGCAT
PR-24013	ATCAGTAGCUAGAGACCGGGTTGGCGGCG
PR-24014	ACTTTTTGCAGTACUAACCGCAGGACTACATCATTTCGGCGCC
PR-24015	CACGCGAUCTAATGGGTCCAGGGACCG
PR-24016	CGTGCGAUTTAAGAGGGCATAGCAATGGCC

PR-24017	ATCTGTCAUGCCACAATGTCTCAGACCCACAAGCACG
PR-24018	ACTTTTTGCAGTACUAACCGCAGTCAGCGAAATCCATTCACGAG
PR-24019	CACGCGAUTTAAACTCCGAGAGGAGTGGAA
PR-24020	CGTGCGAUCTACTTGATCCACCGCCGAA
PR-24021	ATCTGTCAUGCCACAATGACGACGTCTTACAGCGA
PR-24022	ACTTTTTGCAGTACUAACCGCAGTCCAAGGCGAAATTCGAAAGC
PR-24023	CACGCGAUCTACTTCTGTCGCTTGTAAATCTT
PR-24024	AGGCAAAACGCCUGCAGCAGCTCAATCAGCCA
PR-24025	AGGCGTTTTGCCUCGTGTCGGACGACATTATGG
PR-24026	AGGGCTGGCCTCGTCGGGUCTTGGACTCATCCATAATGTCGTCCGACACGAGCCA
PR-24027	ACCCGACGAGGCCAGCCCUGCTGGTACCTCAAGCCCAAGGTCGGCATGATTGCCATCT GG
PR-24028	ACTTTTTGCAGTACUAACCGCAGGATTATAACAGCGCGGATTTCA
PR-24029	CACGCGAUTCACTGCGCATCCTCAAAG
PR-24030	ACTTTTTGCAGTACUAACCGCAGTCCTCTTGCATCAACCCCTC
PR-24031	CACGCGAUTTAGCCCTTGGTGCCGGG
PR-24032	ACTTTTTGCAGTACUAACCGCAGCACATGGCTATCCCCATCAAG
PR-24033	CACGCGAUTTACAGCCACTGCTCGAACA
PR-24034	ACTTTTTGCAGTACUAACCGCAGGCCGAGATGTTCAACGGCA
PR-24035	CACGCGAUTTAGGGAATGATGGGCTCGAC
PR-24036	ACTTTTTGCAGTACUAACCGCAGGGAAAACTCATCGAACTGCTCTT
PR-24037	CACGCGAUCTAATCTCTCAGAGGAAACATCTT
PR-24038	CGTGCGAUCTAAGTCAGCTCGCTCCAAATG
PR-24039	ATCTGTCAUGCCACAATGGTCACCCAACAGTCTGC
PR-24044	CGTGCGAUTCAGAAAGCAAGAACACCAACG
PR-24045	ATCTGTCAUGCCACAATGGGAAAAGAACAAGATCAGGA
PR-24046	ACTTTTTGCAGTACUAACCGCAGATGACGGCTCTCGCATATTAC
PR-24047	CACGCGAUTTACTGCCCTTCCCATCCGC
PR-24048	TCCCATTCTGTCGGTAATGTCG
PR-24049	AGGCCACUGGACACAAACTCGCTTGTT
PR-24050	AGAGTTGTGTGTCTCGTGTGG
PR-24055	GGATCTCGTTGCCGCCAAAG
PR-24056	ACTCTGTCGTAUCGTCTTTTTATCCACACTTCAAAT
PR-24057	ATACGACAGAGUTCCAAAAATGGCCAGATCGAT
PR-24058	TATACTCTGGCGGTCGCATG
PR-24059	GACATGCGACAACTAAGACATTTTAGAGCT
PR-24060	TGTCTTAGTTGTCGCATGTCTAACCAACCT
PR-24061	ACTTTTTGCAGTACUAACCGCAGTCTACCCTGCCTATCTCTTCT
PR-24062	CACGCGAUTTACACCACCATGGGGTGC
PR-24070	CACGCGAUTTACTCAATCATTCGGAACTCTG
PR-24365	ACTTTTTGCAGTACUAACCGCAGACTATCGACTCACAATACTACA
PR-24366	ACTCTGTCGTAUAGTATATAGCACCACTCCACC
PR-24581	CCTCGCCTCATTTACTAAGCA

PR-24582	ACGTCGTGAAAUATCGACGTGGGGATAATTGAA
PR-24583	ATTTCACGACGUAATCATATGCACTGGCAAATCT
PR-24584	AGTTTTCCCATGUTGTGGTCGACTTGAGTGAGA
PR-24585	ACATGGGAAAACUCATCGAACTGCTCTTGCAC
PR-24586	GCAACACGTCGGTTCCAAA
PR-24587	AACTTCAGAACAATTCGAGGGTTTTAGAGCT
PR-24588	CCTCGAATTGTTCTGAAGTTTAACCAACCT

Synthetic genes used in this study.

>SeACS

ATGTCTCAGACCCACAAGCACGCTATCCCCGCCAACATTGCCGACCGATGCCTGATC
AACCCCGAGCAGTACGAGACTAAGTACAAGCAGTCTATCAACGACCCCGACACCTT
CTGGGGCGAGCAGGGCAAGATCCTGGACTGGATCACCCCTTACCAGAAGGTCAAGA
ACACCTCTTTCGCTCCCGGCAACGTGTCTATCAAGTGGTACGAGGACGGCACCCTGA
ACCTGGCCGCCAACTGCCTGGACCGACACCTCCAAGAGAACGGCGACCGAACCGCC
ATCATCTGGGAGGGCGACGACACCTCTCAGTCTAAGCACATCTCTTACCGAGAGCTG
CACCGAGATGTGTGCCGATTCGCTAACACCCTGCTGGACCTGGGCATCAAGAAGGG
CGACGTCGTCGCTATCTACATGCCCATGGTGCCCGAGGCCGCCGTGGCCATGCTGGC
CTGCGCTCGAATCGGCGCCGTGCACTCTGTGATCTTCGGCGGCTTCTCGCCCGAGGC
TGTGGCCGGACGAATCATCGACTCCTCTTCTCGACTGGTGATTACCGCCGACGAGGG
CGTGCGAGCCGGCCGATCTATTCCCCTGAAGAAGAACGTCGACGACGCTCTGAAGA
ACCCCAACGTGACCTCTGTCGAGCACGTGATCGTGCTGAAGCGAACCGGCTCTGAC
ATCGACTGGCAAGAGGGCCGAGATCTGTGGTGGCGAGATCTGATCGAGAAGGCTTC
TCCCGAGCACCAGCCTGAGGCCATGAACGCTGAGGACCCTCTGTTCATCCTGTACAC
CTCTGGCTCTACCGGCAAGCCCAAGGGCGTGCTGCACACCACCGGCGGCTACCTGGT
GTACGCCGCCACCACCTTCAAGTACGTGTTCGACTACCATCCTGGCGACATCTACTG
GTGCACCGCTGACGTCGGCTGGGTGACCGGCCACTCTTACCTGCTGTACGGACCCCT
GGCCTGTGGCGCTACCACTCTGATGTTCGAGGGCGTCCCCAACTGGCCCACTCCTGC
TCGAATGTGCCAGGTGGTGGACAAGCACCAGGTGAACATTCTGTACACCGCTCCTAC
CGCCATTCGAGCCCTGATGGCCGAGGGCGACAAGGCCATCGAGGGCACCGACCGAT
CTTCTCTGCGAATCCTGGGCTCTGTGGGCGAGCCTATTAACCCCGAGGCCTGGGAGT
GGTACTGGAAGAAGATTGGCAAGGAAAAGTGCCCCGTCGTTGACACCTGGTGGCAG
ACCGAGACTGGCGGCTTCATGATTACCCCTCTGCCTGGCGCCATCGAGCTGAAGGCC
GGCTCTGCTACCCGACCATTCTTCGGCGTGCAGCCCGCTCTGGTCGACAACGAGGGA
CACCCTCAAGAGGGCGCCACCGAGGGCAACCTGGTCATCACCGACTCTTGGCCCGG
ACAGGCTCGAACCCTGTTCGGCGACCACGAGCGATTTGAGCAGACCTACTTCTCTAC
CTTCAAGAACATGTACTTCTCTGGCGACGGCGCTCGACGAGATGAGGACGGCTACTA
CTGGATTACCGGCCGAGTGGACGACGTGCTGAACGTGTCTGGCCACCGACTGGGCA
CCGCCGAGATCGAGTCTGCCCTGGTGGCTCACCCCAAGATCGCCGAGGCTGCCGTCG
TGGGCATTCCCCACGCCATCAAGGGCCAAGCCATCTACGCCTACGTGACCCTCAACC
ACGGCGAGGAACCCTCGCCTGAGCTGTACGCCGAGGTGCGAAACTGGGTGCGAAAG
GAAATCGGTCCCCTGGCTACCCCTGACGTCCTGCATTGGACCGACTCGCTGCCCAAG

ACACGATCTGGAAAGATCATGCGACGAATCCTGCGAAAGATCGCTGCCGGTGACAC
CTCTAACCTGGGCGACACTTCTACCCTGGCTGACCCTGGCGTGGTCGAGAAGCCCCT
GGAAGAGAAGCAGGCCATTGCTATGCCCTCTTAA

>ClLS

ATGTCCTCTTGCATCAACCCCTCTACTCTGGTGACCTCTGTGAACGCCTTCAAGTGTC
TGCCCCTGGCCACCAACAAGGCCGCCATCCGAATCATGGCCAAGTACAAGCCCGTG
CAGTGCCTGATCTCTGCCAAGTACGACAACCTGACCGTGGACCGACGATCTGCCAAC
TACCAGCCTTCTATCTGGGACCACGACTTTCTGCAGTCTCTGAACTCTAACTACACC
GACGAGGCCTACAAGCGACGAGCCGAGGAACTGCGAGGCAAGGTGAAGATCGCCA
TCAAGGACGTGATCGAGCCCCTGGACCAGCTGGAACTGATTGACAACCTGCAGCGA
CTGGGACTCGCCCACCGATTCGAGACTGAGATCCGAAACATCCTGAACAACATCTAT
AACAACAACAAGGACTACAACTGGCGAAAGGAAAACCTGTACGCCACCTCTCTCGA
GTTCCGACTGCTGCGACAGCACGGCTACCCCGTGTCTCAAGAGGTGTTCAACGGCTT
CAAGGACGACCAAGGCGGCTTCATCTGCGACGACTTCAAGGGCATCCTGTCTCTGCA
CGAGGCCTCTTACTACTCGCTCGAGGGCGAGTCTATCATGGAAGAGGCCTGGCAGTT
CACCTCTAAGCACCTGAAGGAAGTGATGATCTCTAAGAACATGGAAGAAGATGTGT
TCGTCGCCGAGCAGGCCAAGCGAGCCCTGGAACTGCCCCTGCACTGGAAGGTGCCC
ATGCTCGAGGCCCGATGGTTCATCCACATCTACGAACGACGAGAGGACAAGAACCA
CCTCCTGCTCGAGCTGGCCAAGATGGAATTCAACACCCTGCAGGCCATCTACCAAGA
GGAACTGAAGGAAATCTCTGGCTGGTGGAAGGACACCGGCCTGGGCGAGAAGCTGT
CTTTCGCCCGAAACCGACTGGTGGCCTCTTTCCTGTGGTCTATGGGAATCGCCTTCGA
GCCCCAGTTCGCCTACTGCCGACGAGTGCTGACCATCTCTATCGCCCTGATCACCGT
GATCGACGATATCTACGACGTGTACGGCACCCTGGACGAGCTTGAGATTTTCACCGA
CGCCGTGGAACGATGGGACATCAACTACGCCCTGAAGCACTTGCCCGGCTATATGA
AGATGTGCTTCCTGGCTCTGTACAACTTCGTGAACGAGTTCGCTTACTACGTGCTGA
AGCAGCAGGACTTCGACCTGCTGCTGTCTATCAAGAACGCCTGGCTGGGCCTGATCC
AGGCCTACCTGGTCGAGGCCAAGTGGTATCACTCCAAGTACACCCCTAAGCTGGAA
GAGTACCTCGAGAACGGCCTGGTGTCTATCACCGGACCTCTGATCATCACCATCTCT
TACCTGTCTGGCACTAACCCCATCATCAAGAAGGAACTGGAATTCCTCGAGTCTAAC
CCCGACATCGTGCACTGGTCCTCTAAGATCTTTCGACTGCAGGACGACCTGGGCACC
TCTTCTGACGAGATCCAGCGAGGCGACGTGCCCAAGTCTATCCAGTGCTACATGCAC
GAGACTGGCGCCTCTGAGGAAGTGGCCCGACAGCACATTAAGGACATGATGCGACA
GATGTGGAAGAAGGTCAACGCCTATACCGCCGACAAGGACTCTCCCCTGACCGGAA
CCACCACCGAGTTTCTGCTGAACCTGGTGCGAATGTCTCACTTCATGTACCTGCACG
GTGACGGCCACGGCGTGCAAAACCAAGAGACTATCGACGTGGGCTTCACCCTGCTG
TTCCAGCCTATTCCTCTCGAGGACAAGCACATGGCCTTCACCGCTTCTCCCGGCACC
AAGGGCTAA

>PfLS

ATGCACATGGCTATCCCCATCAAGCCCGCTCACTACCTGCACAACTCTGGCCGATCT
TACGCCTCTCAGCTGTGCGGCTTCTCTTCTACCTCTACTCGAGCCGCCATTGCTCGAC
TGCCCCTGTGCCTGCGATTCCGATGCTCTCTGCAGGCCTCTGACCAGCGACGATCTG
GCAACTACTCTCCCTCTTTCTGGAACGCCGACTACATCCTGTCTCTGAACTCTCACTA
CAAGGACAAGTCTCACATGAAGCGAGCCGGCGAGCTGATCGTGCAGGTCAAGATGG
TGATGGGCAAGGAAACTGACCCCGTCGTCCAGCTTGAGCTGATTGACGACCTGCAG
AAGCTGGCCCTGTCTCACCACGTCGAGAAGGAAATCAAGGAAATTCTGTTCAAGAT
CTCTACCTACGACCACAAGATCATGGTCGAGCGAGATCTGTACTCTACCGCTCTGGC
CTTCCGACTGCTGCGACAGTACGGCTTCAAGGTGCCCCAAGAGGTGTTCGACTGCTT
CAAGAACGACAACGGCGAGTTCAAGCGATCTCTGTCCTCTGACACCAAGGGCCTGC
TGCAGCTGTACGAGGCTTCGTTCCTGCTGACCGAGGGCGAGATGACCCTCGAGCTGG
CCCGAGAGTTCGCCACCAAGTCTCTGCAAGAGAAGCTGAACGAAAAGACCATTGAC
GACGACGATGACGCCGACACCAACCTGATCTCTTGCGTGCGACACTCTCTGGACATC
CCCATCCACTGGCGAATTCAGCGACCCAACGCCTCTTGGTGGATCGACGCCTACAAG
CGAAGATCCCACATGAACCCTCTGGTGCTGGAACTGGCTAAGCTGGACCTGAACAT
CTTCCAGGCTCAGTTTCAGCAAGAGCTGAAGCAGGACCTCGGCTGGTGGAAGAACA
CCTGTCTGGCCGAGAAGCTGCCCTTCGTGCGAGATCGACTGGTCGAGTGCTACTTCT
GGTGCACCGGCATCATTCAGCCCCTGCAGCACGAGAACGCCCGAGTGACCCTGGCC
AAGGTGAACGCCCTGATCACCACTCTGGACGACATCTACGACGTGTACGGCACCCTC
GAGGAACTCGAGCTGTTCACCGAGGCCATCCGACGATGGGACGTGTCCTCTATCGA
CCATCTGCCTAACTATATGCAGCTGTGCTTTCTGGCTCTGAACAACTTCGTGGACGA
CACCGCCTACGACGTCATGAAGGAAAAGGACATCAACATCATTCCCTACCTGCGAA
AGTCTTGGCTGGACCTCGCCGAGACTTACCTGGTCGAGGCCAAGTGGTTCTACTCTG
GACACAAGCCCAACCTGGAAGAGTACCTGAACAACGCCTGGATCTCTATCTCTGGC
CCCGTGATGCTGTGCCACGTGTTCTTTCGAGTGACCGACTCTATCACCCGAGAGACT
GTCGAGTCCCTGTTCAAGTACCACGACCTGATCCGATACTCCTCTACCATCCTGCGA
CTGGCCGACGACCTGGGCACCTCTCTGGAAGAGGTGTCCCGAGGCGACGTGCCCAA
GTCTATCCAGTGCTACATGAACGATAACAACGCTTCTGAGGAAGAGGCCCGACGAC
ACATCCGATGGCTGATCGCTGAGACTTGGAAGAAGATCAACGAAGAGGTCTGGTCT
GTGGACTCGCCCTTCTGCAAGGACTTCATTGCCTGTGCCGCCGACATGGGCCGAATG
GCCCAGTTCATGTACCACAACGGTGACGGCCACGGCATTCAGAACCCTCAGATCCA
CCAGCAGATGACCGACATCCTGTTCGAGCAGTGGCTGTAA

>AaBFS

ATGTCTACCCTGCCTATCTCTTCTGTGTCCTCGTCTTCGTCTACCTCTCCTATCGTGGT
GGACGACAAGGACTCTACCAAGCCTGACGTGATCCGACACACCACCAACTTCAACG
CCTCTATCTGGGGCGACCAGTTCCTGACCTACGACGAGCCCGAGGACCTGGTGATGA
AGAAGCAGCTGGTCGAGGAACTGAAGGAAGAGGTCAAGAAGGAACTGATCACCAT
CAAGGGCTCTAACGAGCCCATGCAGCACGTGAAGCTGATCGAGCTGATTGACGCCG
TGCAGCGACTGGGAATCGCCTACCACTTCGAGGAAGAGATCGAGGAAGCTCTGCAG
CACATCCACGTCACCTACGGCGAGCAGTGGGTCGACAAGGAAAACCTGCAGTCTAT
CTCCCTGTGGTTCCGACTGCTGCGACAGCAGGGCTTCAACGTGTCCTCTGGCGTGTT
CAAGGACTTCATGGACGAGAAGGGCAAGTTCAAGGAATCTCTGTGCAACGACGCCC

AGGGCATTCTGGCCCTGTACGAGGCCGCCTTCATGCGAGTCGAGGACGAGACTATC
CTGGACAACGCCCTCGAGTTCTCTAAGGTCCACCTGGACATCATTGCTAAGGACCCC
TCTTGCGACTCTTCGCTGCGAACCCAGATCCACCAGGCTCTGAAGCAGCCCCTGCGA
CGACGACTGGCCCGAATCGAGGCCCTGCACTACATGCCCATCTACCAGCAAGAGAC
TTCTCACGACGAGGTGCTGCTGAAGCTGGCCAAGCTGGACTTCTCCGTGCTGCAGTC
CATGCACAAGAAGGAATTGTCTCACATCTGCAAGTGGTGGAAGGACCTGGACCTGC
AGAACAAGCTGCCCTTCGTCCGAGATCGAGTGGTCGAGGGCTACTTCTGGATCCTGT
CTATCTACTACGAGCCCCAGCACGCTCGAACCCGAATGTTCCTGATGAAGTCTTGCA
TGTGGCTGGTGGTGCTGGACGACACCTTCGACAACTACGGCACCTACGAGGAACTC
GAGATCTTCACCCAGGCCGTCGAGAAGTGGTCTATCTCTTGCCTGGACATGCTGCCC
GAGTACATGAAGCTCATCTACCAAGAGCTGGTGAACCTGCACGTCGAGATGGAAGA
GTCTCTCGAGAAGGAAGGCAAGGCCTACCAGATTCACTACGTGAAGGAAATGGCCA
AGGAACTCGTGCGAAACTACCTGGTTGAGGCCCGATGGCTCAAGGAAGGCTACATG
CCTACTCTGGAAGAGTACATGTCTGTGTCTATGGTGACCGGAACCTACGGCCTGATG
ACCGCTCGATCTTACGTCGGCCGAGGCGACATCGTGAACGAGGACACCTTCAAGTG
GGTGTCGTCTTACCCTCCAATTGTGAAGGCCTCTTGCGTGATCATCCGACTGATGGA
CGACATTGTGTCTCACAAGGAAGAACAAGAGCGAGGCCACGTCGCCTCTTCTATCG
AGTGCTACTCTAAGGAATCTGGCGCCTCTGAGGAAGAGGCCTGCGAGTACATCTCTC
GAAAGGTTGAGGACGCCTGGAAGGTGATCAACCGAGAGTCTCTGCGACCCACCGCT
GTGCCCTTTCCACTGCTGATGCCCGCCATCAACCTGGCTCGAATGTGTGAGGTGCTG
TACTCCGTGAACGACGGCTTCACCCACGCTGAGGGCGACATGAAGAGTTACATGAA
GTCGTTCTTCGTGCACCCCATGGTGGTGTAA

>CnVS

ATGGCCGAGATGTTCAACGGCAACTCTTCTAACGACGGATCTTCTTGCATGCCCGTG
AAGGACGCCCTGCGACGAACCGGCAACCACCATCCTAACCTGTGGACCGACGACTT
CATCCAGTCTCTGAACTCTCCCTACTCTGACTCTTCTTACCACAAGCACCGAGAGAT
CCTGATCGACGAGATCCGAGACATGTTCTCTAACGGCGAGGGCGACGAGTTCGGCG
TGCTCGAGAACATCTGGTTCGTGGACGTGGTGCAGCGACTGGGCATCGACCGACACT
TCCAAGAGGAAATCAAGACCGCTCTGGACTACATCTACAAGTTCTGGAACCACGAC
TCTATCTTCGGCGACCTGAACATGGTGGCCCTGGGCTTCCGAATCCTGCGACTCAAC
CGATACGTGGCCTCTTCTGACGTGTTCAAGAAGTTCAAGGGCGAAGAGGGCCAGTTC
TCTGGCTTCGAGTCCTCTGACCAGGACGCCAAGCTCGAGATGATGCTGAACCTGTAC
AAGGCCTCTGAGCTGGACTTCCCCGACGAGGACATCCTGAAGGAAGCCCGAGCCTT
CGCCTCTATGTACCTGAAGCACGTGATCAAGGAATACGGCGACATCCAAGAGTCTA
AGAACCCTCTGCTGATGGAAATCGAGTACACCTTCAAGTACCCCTGGCGGTGTCGAC
TGCCCCGACTCGAGGCCTGGAACTTCATTCACATCATGCGACAGCAGGACTGCAAC
ATCTCTCTGGCCAACAACCTCTACAAGATCCCCAAGATCTACATGAAGAAGATTCTC
GAGCTGGCCATCCTGGACTTCAACATCCTGCAGTCTCAGCACCAGCACGAGATGAA
GCTCATTTCTACCTGGTGGAAGAACTCTTCTGCTATCCAGCTCGACTTCTTCCGACAC
CGACACATCGAGTCTTACTTTTGGTGGGCTTCGCCCCTGTTCGAGCCCGAGTTCTCTA
CCTGCCGAATCAACTGCACCAAGCTGTCTACCAAGATGTTCCTGCTCGACGACATCT
ACGACACCTACGGCACCGTCGAGGAACTGAAGCCCTTCACCACCACTCTGACCCGA

TGGGACGTGTCTACCGTGGACAACCATCCTGACTATATGAAGATCGCCTTCAACTTC
TCTTACGAGATCTACAAGGAAATCGCCTCTGAGGCCGAGCGAAAGCACGGCCCCTT
CGTGTACAAGTACCTGCAGTCGTGCTGGAAGTCTTACATCGAGGCCTACATGCAAGA
GGCCGAGTGGATCGCCTCTAACCACATTCCTGGATTCGACGAGTACCTGATGAACGG
CGTGAAGTCCTCTGGCATGCGAATCCTGATGATTCACGCCCTGATCCTGATGGACAC
CCCTCTGTCTGACGAGATTCTGGAACAGCTGGACATCCCTTCGTCTAAGTCTCAGGC
CCTGCTGTCTCTGATCACCCGACTGGTGGACGACGTCAAGGACTTCGAGGACGAGC
AGGCCCACGGCGAGATGGCCTCGTCTATCGAGTGTTACATGAAGGACAACCACGGC
TCTACCCGAGAGGACGCTCTGAACTACCTGAAGATCCGAATCGAGTCCTGCGTGCA
AGAGCTGAACAAGGAACTGCTCGAGCCCTCTAACATGCACGGATCTTTCCGAAACC
TGTACCTCAACGTGGGAATGCGAGTGATTTTCTTCATGCTGAACGACGGTGACCTGT
TCACCCACTCTAACCGAAAGGAAATCCAGGACGCTATCACCAAGTTCTTCGTCGAGC
CCATCATTCCCTAA

>XdCrtl

ATGGGAAAAGAACAAGATCAGGATAAACCCACAGCTATCATCGTGGGATGTGGTAT
CGGTGGAATCGCCACTGCCGCTCGTCTTGCTAAAGAAGGTTTCCAGGTCACGGTGTT
CGAGAAGAACGACTACTCCGGAGGTCGATGCTCTTTAATCGAGCGAGATGGTTATC
GATTCGATCAGGGGCCCAGTTTGCTGCTCTTGCCAGATCTCTTCAAGCAGACATTCG
AAGATTTGGGGGAGAAGATGGAAGATTGGGTCGATCTCATCAAGTGTGAACCCAAC
TATGTTTGCCACTTCCACGATGAAGAGACTTTCACTTTTTCAACCGACATGGCGTTGC
TCAAGCGGGAAGTCGAGCGTTTTGAAGGCAAAGATGGATTTGATCGGTTCTTGTCGT
TTATCCAAGAAGCCCACAGACATTACGAGCTTGCTGTCGTTCACGTCCTGCAGAAGA
ACTTCCCTGGCTTCGCAGCATTCTTACGGCTACAGTTCATTGGCCAAATCCTGGCTCT
TCACCCCTTCGAGTCTATCTGGACAAGAGTTTGTCGATATTTCAAGACCGACAGATT
ACGAAGAGTCTTCTCGTTTGCAGTGATGTACATGGGTCAAAGCCCATACAGTGCGCC
CGGAACATATTCCTTGCTCCAATACACCGAATTGACCGAGGGCATCTGGTATCCGAG
AGGAGGCTTTTGGCAGGTTCCTAATACTCTTCTTCAGATCGTCAAGCGCAACAATCC
CTCAGCCAAGTTCAATTTCAACGCTCCAGTTTCCCAGGTTCTTCTCTCTCCTGCCAAG
GACCGAGCGACTGGTGTTCGACTTGAATCCGGCGAGGAACATCACGCCGATGTTGT
GATTGTCAATGCTGACCTCGTTTACGCCTCCGAGCACTTGATTCCTGACGATGCCAG
AAACAAGATTGGCCAACTGGGTGAAGTCAAGAGAAGTTGGTGGGCTGACTTAGTTG
GTGGAAAGAAGCTCAAGGGAAGTTGCAGTAGTTTGAGCTTCTACTGGAGCATGGAC
CGAATCGTGGACGGTCTGGGCGGACACAATATCTTCTTGGCCGAGGACTTCAAGGG
ATCATTCGACACAATCTTCGAGGAGTTGGGTCTCCCAGCCGATCCTTCCTTTTACGTG
AACGTTCCCTCGCGAATCGATCCTTCTGCCGCTCCCGAAGGCAAAGATGCTATCGTC
ATTCTTGTGCCGTGTGGCCATATCGACGCTTCGAACCCTCAAGATTACAACAAGCTT
GTTGCTCGGGCAAGGAAGTTTGTGATCCAAACGCTTTCCGCCAAGCTTGGACTTCCC
GACTTTGAAAAAATGATTGTGGCAGAGAAGGTTCACGATGCTCCCTCTTGGGAGAA
AGAATTTAACCTCAAGGACGGAAGCATCTTGGGACTGGCTCACAACTTTATGCAAGT
TCTTGGTTTCAGGCCGAGCACCAGACATCCCAAGTATGACAAGTTGTTCTTTGTCGG
GGCTTCGACTCATCCCGGAACTGGGGTTCCCATCGTCTTGGCTGGAGCCAAGTTAAC
TGCCAACCAAGTTCTCGAATCCTTTGACCGATCCCCAGCTCCAGATCCCAATATGTC

ACTCTCCGTACCATATGGAAAACCTCTCAAATCAAATGGAACGGGTATCGATTCTCA
GGTCCAGCTGAAGTTCATGGATTTGGAGAGATGGGTATACCTTTTGGTGTTGTTGAT
TGGGGCCGTGATCGCTCGATCCGTTGGTGTTCTTGCTTTCTGA

>XdCtrYB

ATGACGGCTCTCGCATATTACCAGATCCATCTGATCTATACTCTCCCAATTCTTGGTC
TTCTCGGCCTGCTCACTTCCCCGATTTTGACAAAATTTGACATCTACAAAATATCGAT
CCTCGTATTTATTGCGTTTAGTGCAACCACACCATGGGACTCATGGATCATCAGAAA
TGGCGCATGGACATATCCATCAGCGGAGAGTGGCCAAGGCGTGTTTGGAACGTTTCT
AGATGTTCCATATGAAGAGTACGCTTTCTTTGTCATTCAAACCGTAATCACCGGCTT
GGTCTACGTCTTGGCAACTAGGCACCTTCTCCCATCTCTCGCGCTTCCCAAGACTAG
ATCGTCCGCCCTTTCTCTCGCGCTCAAGGCGCTCATCCCTCTGCCCATTATCTACCTA
TTTACCGCTCACCCCAGCCCATCGCCCGACCCGCTCGTGACAGATCACTACTTCTAC
ATGCGGGCACTCTCCTTACTCATCACCCCACCTACCATGCTCTTGGCAGCATTATCA
GGCGAATATGCTTTCGATTGGAAAAGTGGCCGAGCAAAGTCAACTATTGCAACAAT
CATGATCCCGACGGTGTATCTGATTTGGGTAGATTATGTTGCTGTCGGTCAAGACTC
TTGGTCGATCAACGATGAGAAGATTGTAGGGTGGAGGCTTGGAGGTGTACTACCCA
TTGAGGAAGCTATGTTCTTCTTACTGACGAATCTAATGATTGTTCTGGGTCTGTCTGC
CTGCGATCATACTCAGGCCCTATACCTGCTACACGGTCGAACTATTTATGGCAACAA
AAAGATGCCATCTTCATTTCCCCTCATTACACCGCCTGTGCTCTCCCTGTTTTTTAGC
AGCCGACCATACTCTTCTCAGCCAAAACGTGACTTGGAACTGGCAGTCAAGTTGTTG
GAGGAAAAGAGCCGGAGCTTTTTTGTTGCCTCGGCTGGATTTCCTAGCGAAGTTAGG
GAGAGGCTGGTTGGACTATACGCATTCTGCCGGGTGACTGATGATCTTATCGACTCT
CCTGAAGTATCTTCCAACCCGCATGCCACAATTGACATGGTCTCCGATTTTCTTACCC
TACTATTTGGGCCCCCGCTACACCCTTCGCAACCTGACAAGATCCTTTCTTCGCCTTT
ACTTCCTCCTTCGCACCCTTCCCGACCCACGGGAATGTATCCCCTCCCGCCTCCTCCT
TCGCTCTCGCCTGCCGAGCTCGTTCAATTCCTTACCGAAAGGGTTCCCGTTCAATACC
ATTTCGCCTTCAGGTTGCTCGCTAAGTTGCAAGGGCTGATCCCTCGATACCCACTCG
ACGAACTCCTTAGAGGATACACCACTGATCTTATCTTTCCCTTATCGACAGAGGCAG
TCCAGGCTCGGAAGACGCCTATCGAGACCACAGCTGACTTGCTGGACTATGGTCTAT
GTGTAGCAGGCTCAGTCGCCGAGCTATTGGTCTATGTCTCTTGGGCAAGTGCACCAA
GTCAGGTCCCTGCCACCATAGAAGAAAGAGAAGCTGTGTTAGTGGCAAGCCGAGAG
ATGGGAACTGCCCTTCAGTTGGTGAACATTGCTAGGGACATTAAAGGGGACGCAAC
AGAAGGGAGATTTTACCTACCACTCTCATTCTTTGGTCTTCGGGATGAATCAAAGCT
TGCGATCCCGACTGATTGGACGGAACCTCGGCCTCAAGATTTCGACAAACTCCTCAG
TCTATCTCCTTCGTCCACATTACCATCTTCAAACGCCTCAGAAAGCTTCCGGTTCGAA
TGGAAGACGTACTCGCTTCCATTAGTCGCCTACGCAGAGGATCTTGCCAAACATTCT
TATAAGGGAATTGACCGACTTCCTACCGAGGTTCAAGCGGGAATGCGAGCGGCTTG
CGCGAGCTACCTACTGATCGGCCGAGAGATCAAAGTCGTTTGGAAAGGAGACGTCG
GAGAGAGAAGGACAGTTGCCGGATGGAGGAGAGTACGGAAAGTCTTGAGTGTGGTC
ATGAGCGGATGGGAAGGGCAGTAA

References:

Angerer, H., Radermacher, M., Ma kowska, M., Steger, M., Zwicker, K., Heide, H., et al. (2014). The LYR protein subunit NB4M/NDUFA6 of mitochondrial complex I anchors an acyl carrier protein and is essential for catalytic activity. *Proc. Natl. Acad. Sci.* 111, 5207–5212. doi:10.1073/pnas.1322438111.

Holkenbrink, C., Dam, M. I., Kildegaard, K. R., Beder, J., Dahlin, J., Doménech Belda, D., et al. (2018). EasyCloneYALI: CRISPR/Cas9-Based Synthetic Toolbox for Engineering of the Yeast *Yarrowia lipolytica*. *Biotechnol. J.* 13, 1700543. doi:10.1002/biot.201700543.

Kildegaard, K. R., Adiego-Pérez, B., Doménech Belda, D., Khangura, J. K., Holkenbrink, C., and Borodina, I. (2017). Engineering of *Yarrowia lipolytica* for production of astaxanthin. *Synth. Syst. Biotechnol.* 2, 287–294. doi:10.1016/j.synbio.2017.10.002.

Marella, E. R., Dahlin, J., Dam, M. I., ter Horst, J., Christensen, H. B., Sudarsan, S., et al. (2019). A single-host fermentation process for the production of flavor lactones from non-hydroxylated fatty acids. *Metab. Eng.*, 0–1. doi:10.1016/j.ymben.2019.08.009.
